# On the performance of active RIS-enhanced NOMA systems with spectrum sharing mechanisms

**DOI:** 10.1371/journal.pone.0336951

**Published:** 2025-11-25

**Authors:** Minh Tran, Minh Bui Vu, Sang Quang Nguyen

**Affiliations:** 1 Advanced Intelligent Technology Research Group, Faculty of Electrical and Electronics Engineering, Ton Duc Thang University, Ho Chi Minh City, Vietnam; 2 Faculty of Engineering and Technology, Nguyen Tat Thanh University, Ho Chi Minh City, Vietnam; 3 Posts and Telecommunications Institute of Technology, Ho Chi Minh City, Vietnam; Ozyegin University: Ozyegin Universitesi, TÜRKIYE

## Abstract

This paper presents a comprehensive performance analysis of a downlink non-orthogonal multiple access (NOMA) network assisted by an active reconfigurable intelligent surface (ARIS) in a cognitive spectrum-sharing scenario. Unlike conventional passive RIS (PRIS), the ARIS can both adjust phase shifts and amplify incident signals, thereby mitigating inter-user interference and overcoming multiplicative fading. We consider a two-user secondary network coexisting with a primary user, where the base station communicates with the secondary users via the ARIS. Closed-form expressions for the outage probability (OP), throughput, energy efficiency (EE), and an approximation for the ergodic data rate (EDR) are derived under Nakagami-*m* fading, along with asymptotic OP analysis to reveal the achievable diversity order. We also formulate and solve an optimization problem for the NOMA power allocation coefficient to minimize OP. The main contributions are: (i) proposing an ARIS-assisted NOMA architecture for spectrum-sharing networks and comparing it with PRIS and orthogonal multiple access (OMA) schemes; (ii) deriving exact OP, throughput, and EE expressions, and an approximate EDR expression, validated by Monte Carlo simulations; (iii) providing asymptotic OP analysis to characterize the diversity order; and (iv) optimizing NOMA power allocation to minimize OP. Numerical results confirm that ARIS significantly outperforms PRIS and OMA in terms of OP, throughput, EC, and EE, demonstrating its potential to enhance spectral efficiency, reliability, and coverage in next-generation spectrum-sharing NOMA networks.

## 1 Introduction

The non-orthogonal multiple access (NOMA) has become a potential multiple access contender for sixth-generation wireless networks, which need to fulfill stringent standards including low latency, high spectrum efficiency, and massive connectivity [1,2]. Essentially, NOMA’s capacity to accommodate many users through a single resource block greatly boosts spectral efficiency. In contrast, the conventional orthogonal multiple access (OMA) system assigns distinct users to distinct resource blocks (time, frequency, and code). However, in power-domain (PD-NOMA), users are assigned different power levels, and the base station (BS) sends out a single message that aggregates all of the users’ signals. In order to eliminate inter-user interference and retrieve the broadcast symbol, the receiver side employs successful interference cancellation (SIC) [3]. A controllable analysis framework was presented by the authors of [4] to evaluate the security and reliability of underlay cognitive radio networks (CRs) that employ non-orthogonal multiple access (NOMA), where a secondary BS broadcasts private information to multiple uniformly distributed secondary users while an external eavesdropper is nearby. Two NOMA users connect via an IoT access point relay using the decode-and-forward (DF) protocol in an Internet-of-things (IoT) network with two-way relaying and non-orthogonal multiple access enabled, according to the study in [5]. An accurate OP prediction and low-computational-complexity deep-learning solution have been developed for a real-time IoT network setup. Several papers delve into the performance analysis of NOMA in diverse scenarios. [6] investigated the performance of a dual-hop mixed Radio Frequency (RF) and Free-Space Optical (FSO) system combined with NOMA. [7] analyzed the ergodic rate and effective capacity of RIS-assisted NOMA networks over Nakagami-m fading channels. [8] provided a performance analysis of a NOMA-based hybrid satellite-terrestrial relay system using millimeter-wave technology. [9] analyzed the performance of multi-hop full-duplex NOMA systems with imperfect interference cancellation and near-field path loss. The integration of RIS technology with NOMA is a recurring theme. [10] explored the energy/rate-reliability trade-offs and rate adaptation in hybrid active-passive STAR-RIS-based NOMA systems. [11] investigated the performance of the active simultaneously transmitting and reflecting surface (ASTARS)-aided NOMA network by considering the impact of perfect/imperfect successive interference cancellation. [12] focused on enhancing data rate and energy efficiency in millimeter-wave communications using reconfigurable intelligent surfaces. [13] examines holographic reconfigurable intelligent surface-aided downlink NOMA IoT networks in short-packet communication. [14] investigated the capacity improvement of NOMA networks using multiple aerial intelligent reflecting surfaces. Security aspects are addressed in [15]. [16] analyzed the reliability, security, and covertness of active reconfigurable repeater-assisted NOMA networks in the Internet of Things. delves into the physical layer security analysis for RIS-aided NOMA systems with non-colluding eavesdroppers. Additionally, [5] explored power beacon and NOMA-assisted cooperative IoT networks with co-channel interference. In [17], the authors evaluated the uplink and downlink performance of the energy harvesting NOMA system and investigated and tested the closed-form expressions for OP in a two-user group. The authors of [18] introduced a multi-carrier-based technique that combines transmit diversity with the NOMA protocol to enhance reliability and sum-rate performance. The authors of [19] examined outage performance and improve simultaneous wireless information and power transmission (SWIPT) in a wireless sensor network that harvests energy and uses NOMA. In [20], the authors examined uplink and downlink transmissions in NOMA networks with the use of unmanned aerial vehicles. A large number of other articles have also examined the OP in cooperative relaying systems that use NOMA [21–24]. Although NOMA offers several advantages for communication systems, its ability to solve issues brought on by wireless channel randomization significantly restricts the performance gains it can make.Additionally, one of the physical layer technologies in 6G networks, the reconfigurable intelligent surface (RIS), has been presented [25–30]. It may change the direction of incident electromagnetic waves to the targeted users with variable amplitudes. The passive RIS (PRIS), which is made up of passive reconfigurable parts, has the advantages of low energy consumption and loss in auxiliary wireless communications, which prevents the incident wave’s energy from being increased [26]. In contrast to PRIS, active RIS (ARIS) amplifies incident signals by setting some active reflection components [31]. It is important to note that the abbreviation of ARIS is utilized to make the full-text statements in the following paragraph more brief. It has the ability to overcome the multiplicative fading that PRIS causes. This kind of ARIS offers a novel way to decrease the size of the surface array and improve wireless signal coverage [32]. To improve system capacity and coverage, several theses have so far adopted the PRIS to support NOMA communications [33–35]. PRIS and NOMA function in tandem and have a mutually beneficial effect on 6G networks. The PRIS-assisted NOMA transmission mechanism, which was particularly built by the authors of [33], examined user outage behaviors using an on-off switch. Through the design of PRIS phase shifting, the transmit power at the BS was compared between PRIS-NOMA and PRIS-OMA in [34]. It was revealed in [35] that PRIS-NOMA had a lower OP than conventional relaying methods. PRIS-NOMA’s beamforming architecture was examined with power budget limitations while taking downlink transmission into account [36]. A survey of the different PRIS applications to NOMA systems was conducted in [37], where it was shown that centralized PRIS deployment can increase user diversity. By dividing the PRIS zone, the total rate of PRIS-NOMA was examined [38], which makes the detection SIC program easier for powerful users. The EDR of two paired users for PRIS-NOMA networks employing wireless information power transmission was examined by the authors of [39] from the standpoint of harvested energy. The system total rate of uplink PRIS-NOMA was examined in [40] while accounting for direct connections. In order to mitigate the interference caused by poor SIC, the inventors of [41] also devised the uplink PRIS-NOMA’s equalizers and phase shift. The on-off control approach was used by the authors in [42] to assess the security characteristics of PRIS-NOMA from the perspective of safety applications. Additionally, the power allocation and passive beam were optimized to determine the system’s attainable rates of aerial PRIS-NOMA [43]. Compared to PRIS, many scholars have concentrated on the usage of active RIS (ARIS) in wireless communication [44–46]. Because it can overcome the double route loss from the source to PRIS and subsequently to the destinations, ARIS is superior to PRIS in addressing the double fading impact. The capacity performance boost provided by ARIS was examined by the authors of [47] in order to address these fundamental constraints. In comparison to PRIS, it is shown that the RIS may offer a higher sum rate. According to a study on the attainable rate of an ARIS-assisted downlink system conducted in [48], ARIS should be installed closer to the intended users with a lower amplification power. With the introduction of ARIS to wireless systems, the double path loss generated by PRIS was transformed into the extra form. Compared to PRIS, ARIS has the benefit of system throughput under the same power budget [49]. The energy efficiency of ARIS sub/fully linked structures was also examined by the authors of [50], who demonstrated that the sub-connected structure can lower power usage. In [51], adaptive beamforming was designed to emphasize the weighted capacity of ARIS-aided, wireless-powered systems.The power consumption data are detailed in the hybrid relay/ARIS designs that were built by the authors of [52]. Therefore, in [53], a novel hybrid PRIS/ARIS design was presented with amplification factors restricted. In order to provide insight into radar communication, the authors of [37] examined the likelihood of an ARIS-assisted radar system being detected.Recent research has demonstrated a significant increase in utilizing ARIS with NOMA systems in wireless communications. This trend is driven by ARIS’s impressive advancements and the potential of ARIS-NOMA to enhance the efficiency and performance of wireless networks.Several studies have explored the benefits of ARIS-NOMA. For instance, the authors in [54] investigated the aggregate throughput of uplink ARIS-NOMA, while [55] examined its achievable rate under restricted energy conditions, confirming its superiority over ARIS-OMA. Further research in [56] utilized reinforcement learning to investigate the communication likelihood of an ARIS-aided energy harvesting NOMA system. To address user needs, [57] calculated the aggregate attainable capacity of an active surface-assisted NOMA system.Beyond these benefits, ARIS has also been shown to enhance rate-splitting multiple access [58], offering a balance between computational complexity and resource efficiency. The authors in [59] recently examined the performance of ARIS-NOMA, taking hardware limitations into account. Most recently, [60] analyzed the performance of ARIS-NOMA in secure communications with discrete phase shifting. [61] further investigated the performance of ARIS-NOMA in covert communication with hardware impairments, comparing the performance of active and passive RIS systems.Meanwhile, [62] focused on the secure communication of active RIS-assisted NOMA networks, assuming that each ARIS element owns a dedicated active reflection-type amplifier.These studies highlight that ARIS-NOMA is a promising research area with significant potential for application in next-generation wireless communication. Although research on ARIS-assisted NOMA systems is still in its early stages, existing studies have laid the groundwork for understanding PRIS and ARIS in wireless communications. ARIS, which can be implemented using current-inverting converters or asymmetric current mirrors, offers the potential to improve coverage for obstructed or fading communication links by amplifying reflected signals [54,55]. This advantage comes at the cost of increased power consumption. However, by enabling ARIS to support NOMA, the system’s spectral efficiency can be significantly enhanced. In theory, ARIS-NOMA systems allow for flexible ARIS deployment and expanded coverage for targeted users. Current research on ARIS-NOMA primarily focuses on optimizing the cumulative data rate. For example, [54] investigated the uplink cumulative data rates of ARIS-NOMA with multiple antennas using alternating optimization, while [55] developed a beamforming design to maximize ARIS-NOMA throughput. Despite these advances, there remains a critical need to evaluate ARIS-NOMA systems under practical spectrum-sharing scenarios, where interference management toward primary users is crucial. Moreover, most prior works have emphasized throughput or rate maximization, leaving a gap in understanding the outage performance, reliability, and EDR of ARIS-NOMA in cognitive underlay settings. This gap is particularly important because next-generation wireless networks must not only maximize spectrum efficiency but also ensure robust performance under interference and fading conditions. Specifically, this study addresses this gap by analyzing the impact of ARIS on key performance metrics, including OP, throughput, and EDR, for secondary NOMA users sharing the spectrum with a primary user. Unlike previous work such as [60], which explored ARIS for security enhancement; [61], which investigated covert communication with hardware impairments; or [62], which considered secure communication with a different system model, this research utilizes a unique model with dedicated reflection-type amplifiers for each ARIS element. It emphasizes interference management towards the primary user in a spectrum-sharing scenario. This distinct focus and novel system model contribute to a unique analysis and offer valuable insights into the practical implementation of ARIS in NOMA networks. Table 1 shows the comparison between our work and related papers. The main contributions of this treatise are outlined as follows:

- We proposed the ARIS-assisted NOMA network with spectrum-sharing communications to improve the spectrum efficiency, and ARIS is capable of boosting superposed signals and reflecting them to users and to enhance the performance of the system.
- We derived the exact closed-form expression for OP, throughput, and the approximation expression for EDR of users to evaluate the performance of the proposed system with ARIS and PRIS. To get the deep insight of the proposed system, we derived the asymptotic expression for OP and get the diversity order.

**Table 1:** Comparison of this work with existing studies (✓: considered; ✗: not considered).

Ref./Our work	Cognitive Radio	NOMA	OP	High SNR	EC	Energy Efficiency	Optimization
[4]	✓	✓	✓	✗	✗	✗	✗
[5]	✗	✓	✓	✓	✓	✗	✓
[35]	✗	✓	✓	✓	✓	✓	✗
[39]	✗	✓	✓	✓	✓	✗	✗
[53]	✗	✗	✗	✗	✓	✗	✓
[54]	✗	✓	✗	✗	✓	✗	✓
[59]	✗	✓	✓	✓	✓	✓	✗
Our work	✓	✓	✓	✓	✓	✓	✓

**Organization:** The subsequent sections of this manuscript are organized as follows. Sect 2 delineates the system architecture of ARIS-assisted NOMA spectrum-sharing communications. The exact and approximate outage probabilities pertinent to ARIS-NOMA spectrum-sharing networks are meticulously delineated in Sect 3. The numerical evaluations are presented in Sect 4. Lastly, Sect 5 encapsulates the findings of this manuscript. The mathematical proofs are compiled in the appendix.

**Notation:** The important notations in this work are shown here. The symbol $(\cdot {)}^{H}$ indicates the conjugate transpose operation. Pr(.) denotes the probability operator; E{.} and D{.} denote the expectation and variance operations, respectively; H(x) is so-called the Heaviside step function; Γ(a) is the Gamma function; Γ(.,.) denotes the upper incomplete gamma function; $G_{p,q}^{m,n}\left[.\right]$ is the Meijer G-function; the probability density function (PDF) and the cumulative distribution function (CDF) of a random variable *X* are represented as $F_{X}\left(.\right)$ and $f_{X}\left(.\right)$, respectively. The symbol diag(·) represents a diagonal matrix with only one member.

## 2 System model

### 2.1 System description

**Fig 1:**
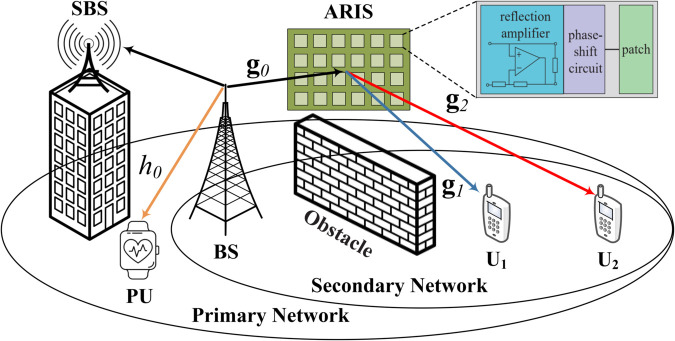
The system model of ARIS-assisted NOMA spectrum-sharing communications, with ARIS capable of boosting superposed signals and reflecting them to users.

We examine a communication scenario involving RIS-assisted NOMA spectrum sharing, illustrated in Fig 1 (The formation of a reduced cluster associated with the two paired devices serviced by BS results in diminished decoding complexity, reduced interference, and minimized latency at the receivers when juxtaposed with the multi-device cluster NOMA [63]. In scenarios where the quantity of devices within the network is significantly high, an escalation in the number of stationary base stations (SBSs) is necessitated, resulting in increased expenses. It is imperative to highlight that the two-user paradigm consistently diminishes latency due to the fewer signal detection processes required between ground users and the SBSs. The presence of a greater number of users within a cluster deteriorates performance for those users due to the substantial interference present among them. Furthermore, the stationary SBS establishes a connected network with adjacent SBSs that are equipped with powered signal processing units capable of efficiently managing multi-device scenarios. It is presupposed that the backhaul connection among SBSs is flawless, and the intricate analytical aspects, as well as the clustering issue, lie beyond the purview of this manuscript.), where a secondary network utilizes the spectrum designated for a primary network that includes a primary user (PU) equipped with a single antenna. The secondary network consists of a single antenna BS serving two single antenna users (the nearby user *U*_1_ and the more distant user *U*_2_) through *N* active reflecting elements. Specifically, contemporary ARIS architectures not only feature identical circuitry for phase shift control as seen in PRIS, but also incorporate power amplifiers to enhance the transmitted signal. The incident superimposed signals at ARIS are boosted with a noticeable gain before being reflected towards the intended users. The inherent multiplicative attenuation present in PRIS-assisted systems will be effectively addressed by active elements while maintaining low power consumption. In practice, we direct our attention to the direct links between BS and users that are hindered by high-rise buildings [64], where the implementation of ARIS can establish a line-of-sight between the BS and non-orthogonal users. Distortions on both the BS and users’ sides are taken into account to more accurately reflect real-world scenarios. We consider a total transmit power budget $P_{max}$ for the secondary BS, which is allocated between NOMA users via predefined power allocation coefficients. The BS–user, BS–PU, BS–ARIS, ARIS–user, and ARIS–PU links are modeled as independent Nakagami-*m* fading channels with shape parameter *m* and average power *λ*, combined with large-scale path loss $d^{-\varsigma }$, where *d* is the link distance and ς is the path loss exponent. This unified channel modeling approach captures both small-scale fading and large-scale attenuation, ensuring that the performance evaluation of ARIS-NOMA spectrum-sharing networks is based on realistic propagation conditions. To represent genuine channel circumstances, we assume that the wireless connections of ARIS-NOMA networks undergo large-scale fading and Nakagami-*m* fading (In our work, we have considered the Nakagami-*m* fading channel, which is widely used to model a broad range of wireless communication environments, including scenarios with severe multipath fading and line-of-sight conditions. The Nakagami-*m* model is a generalization of Rayleigh and Rician fading, making it a more flexible and realistic choice for analyzing RIS-assisted NOMA systems. Moreover, it is worth noting that the derivations of performance analyses specifically designed for ARIS-aided NOMA spectrum-sharing networks can be easily and seamlessly applied across a wide range of multiple fading channels, including Gaussian, Rayleigh, and Rician fading models. This ensures that our analytical framework remains robust and applicable to diverse wireless environments.). Let $g_{i}={\left[g_{i}^{1},\ldots ,g_{i}^{n},\ldots g_{i}^{N}\right]}^{H}$, i∈{1,2} and $g_{0}={\left[g_{0}^{1},\ldots ,g_{0}^{n},\ldots g_{0}^{N}\right]}^{H}$ represent the baseband channel coefficients between ARIS and *U*_*i*_ and BS to the ARIS. $g_{0}^{n}$ and $g_{i}^{n}$ represent the complicated channel factors that exist between BS and ARIS’s *n*th reflective element, as well as between ARIS’s *n*th reflective element and user *i*, respectively. The reflection matrix at ARIS is specified as $\Phi =\sqrt{\beta }\text{diag}\left(e^{j\theta_{1}},\ldots ,e^{j\theta_{n}},\ldots e^{j\theta_{N}}\right)$, where the angle $\theta_{n}$ lies within the interval ranging from 0 to 2π, and the variable *β* signifies the phase shift associated with the *n*th reflection element along with its corresponding reflection amplification coefficient. The reflection elements integrated within ARIS are strategically situated within active amplifiers that utilize either tunneling diodes or resistive converters, thereby enhancing their operational capabilities. Leveraging the fundamental principles of electromagnetic scattering, each individual element is capable of not only reflecting an incoming radio frequency (RF) signal but also amplifying it, with both the amplitude and phase being adjustable to meet specific requirements. This sophisticated feature enables ARIS to attain a reflection amplification coefficient that exceeds unity, which mathematically translates to *β* being greater than 1, as referenced in numerous studies [65,66]. In practical implementations, a negative resistive element, such as a tunnel diode, can be effectively employed to amplify the incident RF signal, facilitating the conversion of direct current bias power into RF power, thereby enhancing the performance of the system. As a result, the integration of such components allows for a remarkable increase in signal strength and quality, which is crucial for advanced communication and imaging applications. Consequently, the innovative design and functionality of ARIS play a pivotal role in advancing the field of radio frequency technology and its myriad applications in both research and industry. We assume that the instantaneous channel state information (CSI) is available at both the BS and ARIS through the use of channel estimation or compressive sensing techniques [67]. The effect of imperfect CSI on the performance of ARIS-NOMA networks will not be addressed in this paper.

### 2.2 Signal Model

In spectrum-sharing systems, the PU requires that the instantaneous interference caused by the BS be less than a set threshold *Q*, which represents the maximum permitted interference power at the PU. At the same time, the BS’ transmit power is limited by a peak power restriction. As a result, the transmit power at the base station may be stated as

$$P_{b}=\min\left({\bar{P}}_{b},\frac{Q}{{\left|h_{0}\right|}^{2}}\right),$$ (1)

where ${\bar{P}}_{b}$ is the maximum transmit power of the BS, *Q* is the interference temperature constraint (ITC) at the PU, and ${\left|h_{0}\right|}^{2}$ represents the power gain of the channel between the BS and PU.

The BS transfers superimposed signals, i.e., $x=\sqrt{a_{1}P_{b}}x_{1}+\sqrt{a_{2}P_{b}}x_{2}$, specifically directed towards user *i* through a process known as ARIS, where the parameters *x*_1_ and *x*_2_ are defined as normalized power signals that maintain a unity value for *U*_1_ and *U*_2_, respectively, ensuring that both users receive the appropriate signal strength. To ensure non-orthogonal users’ fairness, the power allocation factors *a*_1_ and *a*_2_ for *U*_1_ and *U*_2_ must meet the relations $a_{1}<a_{2}$ and $a_{1}\;a{\;}_{2}=1$. *P*_*b*_ represents the BS transmission power. Unlike PRIS, ARIS uses active devices such as tunnel or Gunn diodes to enhance overlaid signals and noise received at itself. In these situations, the received signals at user *i*, (i∈{1,2}) can be described as

$$y_{i}=\sqrt{\beta }g_{i}^{H}\Theta g_{0}x+\sqrt{\beta }g_{i}^{H}\Theta n_{0}+\omega_{i}.$$ (2)

where $\Theta =\text{diag}\left(e^{j\theta_{1}},\ldots ,e^{j\theta_{n}},\ldots e^{j\theta_{N}}\right)$ is so-called the phase shift matrix at ARIS. $n_{0}\sim CN\left(0,N_{tn}I_{N}\right)$ denotes the thermal noise at ARIS with the noise power *N*_*tn*_ and $I_{N}\in {\mathbb{C}}^{N\times 1}$ is the identity matrix. $\omega_{i}$ indicates the Gaussian white noise generated at the user *i* with $\omega_{i}\sim CN\left(0,\sigma^{2}\right)$ in which $\sigma^{2}$ denotes the noise power at user *i*.

In accordance with the NOMA principle, *U*_1_ will initially identify *U*_2_’s information, which has superior channel conditions, and subsequently remove it prior to decoding their own signal. Therefore, the resultant received SINR can be expressed as

$$\gamma_{x_{2}\to x_{1}}=\frac{\beta P_{b}a_{2}{\left|g_{1}^{H}\Theta g_{0}\right|}^{2}}{\beta P_{b}a_{1}{\left|g_{1}^{H}\Theta g_{0}\right|}^{2}+\xi \beta N_{tn}{\left|g_{1}^{H}\Theta \right|}^{2}+\sigma^{2}}\;=\frac{\beta \rho_{b}a_{2}{\left|g_{1}^{H}\Theta g_{0}\right|}^{2}}{\beta \rho_{b}a_{1}{\left|g_{1}^{H}\Theta g_{0}\right|}^{2}+\xi \beta N_{tn}{\left|g_{1}^{H}\Theta \right|}^{2}+1},$$ (3)

Here *ρ*_*b*_ = *P*_*b*_ / *σ*^2^ represents the transmit signal-to-noise ratio (SNR). Parameter ξ denotes the conversion factor between noise and amplified signal power at the ARIS; ξ=1 for ARIS and ξ=0 for PRIS. After eliminating the information from *U*_2_, the receiving SINR for *U*_1_, while decoding its own signal, can be expressed as

$$\gamma_{x_{1}}=\frac{\beta \rho_{b}a_{1}{\left|g_{1}^{H}\Theta g_{0}\right|}^{2}}{\xi \beta N_{tn}{\left|g_{1}^{H}\Theta \right|}^{2}+1}.$$ (4)

Due to the unfavorable channel conditions experienced by *U*_2_, it perceives *x*_1_ as interference and therefore directly identifies its own signal *x*_2_. For now, the SINR that *U*_2_ uses to detect *x*_2_ can be expressed as

$$\gamma_{x_{2}}=\frac{\beta \rho_{b}a_{2}{\left|g_{2}^{H}\Theta g_{0}\right|}^{2}}{\beta \rho_{b}a_{1}{\left|g_{2}^{H}\Theta g_{0}\right|}^{2}+\xi \beta N_{tn}{\left|g_{2}^{H}\Theta \right|}^{2}+1}.$$ (5)

To demonstrate the performance advantages of ARIS-NOMA, we use ARIS-OMA as a to demonstrate the performance advantages of ARIS-NOMA, we use ARIS-OMA as a benchmark for comparison. We have the SINR for user *O* is provided by

$$\gamma_{O}=\frac{\beta \rho_{b}{\left|g_{O}^{H}\Theta g_{0}\right|}^{2}}{\xi \beta N_{tn}{\left|g_{O}^{H}\Theta \right|}^{2}+1}.$$ (6)

where $g_{O}={\left[h_{O}^{1},h_{O}^{2},\ldots ,h_{O}^{n},\ldots ,h_{O}^{N}\right]}^{H}$ denotes the complex channel coefficient between ARIS and user *O*.

## 3 Outage probability

In this segment, we evaluate the performance of ARIS-NOMA/OMA secondary networks by scrutinizing their outage characteristics, wherein we formulate the closed-form expressions for outage probabilities pertaining to *U*_1_ and *U*_2_ within the framework of cascade Nakagami-*m* fading channels. Additionally, we consider the thermal noise produced by ARIS to be a constant in facilitating the computational analysis.

### 3.1 The outage probability of *U*_1_

In this scenario, the occurrence of outage behavior at *U*_1_ transpires when *U*_1_ is unable to perceive the signal from *U*_2_ as well as its own signal. We may articulate such an adverse situation as follows

$$P_{U_{1}}^{ARIS}=1-\mathrm{Pr}\left(\min\left(\frac{\gamma_{x_{2}\to x_{1}}}{\gamma_{th_{2}}},\frac{\gamma_{x_{1}}}{\gamma_{th_{1}}}\right)>1\right)\;=1-\mathrm{Pr}\left(\gamma_{x_{2}\to x_{1}}>\gamma_{th_{2}},\gamma_{x_{1}}>\gamma_{th_{1}}\right),$$ (7)

Here $\gamma_{th_{1}}=2^{R_{1}}-1$ and $\gamma_{th_{2}}=2^{R_{2}}-1$ denote the desired SNRs at *U*_1_ for decoding *x*_1_ and *x*_2_, respectively. *R*_1_ and *R*_2_ represent the corresponding goal rates.

With respect to cascade Nakagami-*m* fading channels, the closed-form for the OP of *U*_1_ within ARIS-NOMA spectrum-sharing networks can be given as

$$\begin{array}{c} P_{U_{1}}^{ARIS}=1-\left[1-e^{-\frac{\mu_{0}\rho_{Q}}{{\bar{\rho }}_{b}}}\sum_{t=0}^{m_{0}-1}\frac{\mu_{0}^{t}\rho_{Q}^{t}}{t!{\bar{\rho }}_{b}^{t}}\right]\frac{\Gamma \left(b_{1}+1,c_{1}^{-1}\sqrt{\frac{\phi_{\max}d_{1}}{{\bar{\rho }}_{b}}}\right)}{\Gamma \left(b_{1}+1\right)}\\ -\frac{2\mu_{0}^{m_{0}}\vartheta_{1}^{-2m_{0}}}{\Gamma \left(m_{0}\right)\Gamma \left(b_{1}+1\right)}\Lambda \left(b_{1},\frac{\mu_{0}}{\vartheta_{1}^{2}},\frac{1}{\vartheta_{1}}\sqrt{\frac{{\bar{\rho }}_{b}}{\rho_{Q}}}\right), \end{array}$$ (8)

where $\phi_{2}=\frac{\gamma_{th_{2}}}{\beta \left(a_{2}-\gamma_{th_{2}}a_{1}\right)}$, $\phi_{1}=\frac{\gamma_{th_{1}}}{\beta a_{1}}$, $\phi_{\max}=\max\left(\phi_{1},\phi_{2}\right)$, $d_{1}=\xi \beta N_{tn}N\lambda_{g_{1}}+1$, $\vartheta_{1}=c_{1}^{-1}\sqrt{\frac{\phi_{\max}d_{1}}{\rho_{Q}}}$ $\mu_{0}=\frac{m_{h_{0}}}{\lambda_{h_{0}}}$, $b_{1}=\frac{N\mu_{1}^{2}}{\Omega_{1}}-1$, $c_{1}=\frac{\Omega_{1}}{\mu_{1}}$, $\mu_{1}=\frac{\Gamma \left(m_{g_{0}}+0.5\right)\Gamma \left(m_{g_{1}}+0.5\right)\sqrt{\lambda_{g_{0}}\lambda_{g_{1}}}}{\Gamma \left(m_{g_{0}}\right)\Gamma \left(m_{g_{1}}\right)\sqrt{m_{g_{0}}m_{g_{1}}}}$, $\Omega_{1}=\lambda_{g_{0}}\lambda_{g_{1}}\left\langle 1-\frac{1}{m_{g_{0}}m_{g_{1}}}{\left[\frac{\Gamma \left(m_{g_{0}}+0.5\right)\Gamma \left(m_{g_{1}}+0.5\right)}{\Gamma \left(m_{g_{0}}\right)\Gamma \left(m_{g_{1}}\right)}\right]}^{2}\right\rangle$, ${\bar{\rho }}_{b}={\bar{P}}_{A}/N_{0}$ denotes the average SNR at the BS and *ρ*_*Q*_ = *Q* / *N*_0_ denotes the average SNR of interference at the PU. We have $m_{h_{0}}$, $m_{g_{0}}$ and $m_{g_{1}}$ denote the Nakagami-*m* fading parameters from the BS–PU, BS–ARIS and ARIS−*U*_1_, respectively. $\lambda_{h_{0}}=E\left\{{\left|h_{0}\right|}^{2}\right\}$, $\lambda_{g_{0}}=E\left\{{\left|g_{0}^{n}\right|}^{2}\right\}$ and $\lambda_{g_{1}}=E\left\{{\left|g_{1}^{n}\right|}^{2}\right\}$ are the mean of ${\left|h_{0}\right|}^{2}$, ${\left|g_{0}^{n}\right|}^{2}$ and ${\left|g_{1}^{n}\right|}^{2}$, respectively. We have E{.} and Γ(.) represent the the expectation operation and the gamma function, respectively. Γ(.,.) denotes the upper incomplete gamma function. $H_{p,q:u,v:e,f}^{m,n:s,t:i,j}\left(\cdot \right)$ represents the extended generalized bivariate Fox H-function (EGBFHF) in [68] and Λ(a,b,c) is shown as

$$\Lambda \left(a,b,c\right)=H_{2,1:0,1:1,1}^{0,2:1,0:0,1}\left(\begin{array}{c} \left(-a-2m_{0};2,1),(1-2m_{0};2,1\right)\\ \left(-2m_{0};2,1\right) \end{array}|\begin{array}{c} -\\ \left(0,1\right) \end{array}|\begin{array}{c} \left(1,1)\right)\\ \left(0,1\right) \end{array}|b,c\right).$$ (9)

In accordance with the definitions pertaining to OP for *U*_1_, the proof procedures commence by integrating (3) and (4) into (7), where $P_{U_{1}}^{ARIS}$ is defined as follows

$$P_{U_{1}}^{ARIS}=1-\mathrm{Pr}\left(\begin{array}{c} {\left|g_{1}^{H}\Theta g_{0}\right|}^{2}>\frac{\phi_{2}}{\rho_{b}}\left(\xi \beta N_{tn}{\left|g_{1}^{H}\Theta \right|}^{2}+1\right),\\ {\left|g_{1}^{H}\Theta g_{0}\right|}^{2}>\frac{\phi_{1}}{\rho_{b}}\left(\xi \beta N_{tn}{\left|g_{1}^{H}\Theta \right|}^{2}+1\right) \end{array}\right)\;=1-\mathrm{Pr}\left({\left|g_{1}^{H}\Theta g_{0}\right|}^{2}>\frac{\phi_{\max}}{\rho_{b}}\left(\xi \beta N_{tn}{\left|g_{1}^{H}\Theta \right|}^{2}+1\right)\right),$$ (10)

where $\phi_{2}=\frac{\gamma_{th_{2}}}{\beta \left(a_{2}-\gamma_{th_{2}}a_{1}\right)}$, $\phi_{1}=\frac{\gamma_{th_{1}}}{\beta a_{1}}$, $\phi_{\max}=\max\left(\phi_{1},\phi_{2}\right)$. It is noted that $\rho_{b}=\min\left({\bar{\rho }}_{b},\frac{\rho_{Q}}{{\left|h_{0}\right|}^{2}}\right)$ in which ${\bar{\rho }}_{b}={\bar{P}}_{b}/\sigma^{2}$ and *ρ*_*Q*_ = *Q* / *σ*^2^, (10) is calculated as

$$P_{U_{1}}^{ARIS}=1-A_{1}-A_{2}.$$ (11)

Here, $A_{1}$ and $A_{2}$ are calculated below as follows

$$A_{1}=\mathrm{Pr}\left(\begin{array}{c} {\left|Z_{1}\right|}^{2}>\frac{\phi_{\max}}{{\bar{\rho }}_{b}}\left(\xi \beta N_{tn}{\left|g_{1}^{H}\Theta \right|}^{2}+1\right),{\left|h_{0}\right|}^{2}<\frac{\rho_{Q}}{{\bar{\rho }}_{b}} \end{array}\right)\;=\mathrm{Pr}\left(\begin{array}{c} \left|Z_{1}\right|>\sqrt{\frac{\phi_{\max}d_{1}}{{\bar{\rho }}_{b}}},{\left|h_{0}\right|}^{2}<\frac{\rho_{Q}}{{\bar{\rho }}_{b}} \end{array}\right)\;=F_{{\left|h_{0}\right|}^{2}}\left(\frac{\rho_{Q}}{{\bar{\rho }}_{b}}\right)\left[1-F_{\left|Z_{1}\right|}\left(\sqrt{\frac{\phi_{\max}d_{1}}{{\bar{\rho }}_{b}}}\right)\right],$$ (12)

and

$$A_{2}=\mathrm{Pr}\left(\begin{array}{c} {\left|g_{1}^{H}\Theta g_{0}\right|}^{2}>\frac{\phi_{\max}{\left|h_{0}\right|}^{2}}{\rho_{Q}}\left(\xi \beta N_{tn}{\left|g_{1}^{H}\Theta \right|}^{2}+1\right),{\left|h_{0}\right|}^{2}>\frac{\rho_{Q}}{{\bar{\rho }}_{b}} \end{array}\right)\;=\mathrm{Pr}\left(\begin{array}{c} \left|Z_{1}\right|>\sqrt{\frac{\phi_{\max}d_{1}{\left|h_{0}\right|}^{2}}{\rho_{Q}}},{\left|h_{0}\right|}^{2}>\frac{\rho_{Q}}{{\bar{\rho }}_{b}} \end{array}\right)\;=\int_{\frac{\rho_{Q}}{{\bar{\rho }}_{b}}}^{\infty }f_{{\left|h_{0}\right|}^{2}}\left(x\right)\left[1-F_{\left|Z_{1}\right|}\left(\sqrt{\frac{\phi_{\max}d_{1}x}{\rho_{Q}}}\right)\right]dx,$$ (13)

where $d_{1}=\xi \beta N_{tn}\lambda_{g_{1}}\;+\;1$, $Z_{i}=g_{i}^{H}\Theta g_{0}=\sum_{n=1}^{N}\left|g_{0}^{n}\right|\left|g_{1}^{n}\right|e^{j\theta_{n}}=\sum_{n=1}^{N}Z_{n}$. It is important to observe that the equation (12) has been formulated with the aid of coherent phase shifting, which serves to optimize the performance received by the intended users. In order to compute equation (12), it is imperative to first determine the PDF and CDF of the random variables $X={\left|h_{0}\right|}^{2}$ and *Z*, respectively. Given that $\left|h_{0}\right|$ is presumed to conform to the Nakagami-*m* distribution, the associated PDF and CDF of *X* is represented as follows

$$f_{{\left|h_{0}\right|}^{2}}\left(x\right)=\frac{\mu_{0}^{m_{0}}x^{m_{0}-1}}{\Gamma \left(m_{0}\right)}e^{-\mu_{0}x},$$ (14a)

$$F_{{\left|h_{0}\right|}^{2}}\left(x\right)=1-\frac{\Gamma \left(m_{0},x\mu_{0}\right)}{\Gamma \left(m_{0}\right)}=1-e^{-\mu_{0}x}\sum_{t=0}^{m_{0}-1}\frac{\mu_{0}^{t}x^{t}}{t!},$$ (14b)

where $\mu_{0}=\frac{m_{h_{0}}}{\lambda_{h_{0}}}$ in which $\lambda_{h_{0}}$ and $m_{h_{0}}$ representing the mean and integer fading factor, respectively. It can be observed that the CDF of *Z* cannot be obtained through a direct method. Nevertheless, we may employ the Laguerre polynomials to furnish an estimated CDF. By considering the optimal phase shift as [69,70], we can obtain $Z_{n}=\left|g_{0}^{n}\right|\left|g_{1}^{n}\right|$ with the expected value and variance of *Z*_*n*_ can be respectively expressed as follows: $\mu_{1}=E\left\{Z_{n}\right\}=\frac{\Gamma \left(m_{g_{0}}+0.5\right)\Gamma \left(m_{g_{1}}+0.5\right)\sqrt{\lambda_{g_{0}}\lambda_{g_{1}}}}{\Gamma \left(m_{g_{0}}\right)\Gamma \left(m_{g_{1}}\right)\sqrt{m_{g_{0}}m_{g_{1}}}}$ and $\Omega_{1}=D\left\{Z_{n}\right\}=\lambda_{g_{0}}\lambda_{g_{1}}\left\langle 1-\frac{1}{m_{g_{0}}m_{g_{1}}}{\left[\frac{\Gamma \left(m_{g_{0}}+0.5\right)\Gamma \left(m_{g_{1}}+0.5\right)}{\Gamma \left(m_{g_{0}}\right)\Gamma \left(m_{g_{1}}\right)}\right]}^{2}\right\rangle$ in which $m_{g_{0}}$, $m_{g_{1}}$ and $m_{g_{2}}$ are the *shape parameter*, indicating the severity of fading and $\lambda_{g_{0}}$, $\lambda_{g_{1}}$ and $\lambda_{g_{2}}$ are the *spread parameter* of the distribution. According to [71,72], let *d*_*PU*_ denote the distance between BS to PU, *d*_*ARIS*_ to denote the distance between BS to ARIS, $d_{U_{i}}$ to denote the distance between ARIS to *U*_*i*_, i=1,2, we get channel gains of fading as $\lambda_{h_{0}}=d_{PU}^{-\varsigma }/d_{0}$, $\lambda_{g_{0}}=d_{ARIS}^{-\varsigma }/d_{0}$, $\lambda_{g_{1}}=d_{U_{1}}^{-\varsigma }/d_{0}$ and $\lambda_{g_{2}}=d_{U_{2}}^{-\varsigma }/d_{0}$ in which ς is the pathloss exponent and *d*_0_ is the reference distance. From [59], the approximate CDF and PDF of *Z* are obtained as

$$f_{Z}\left(x\right)\approx \frac{x^{b_{1}}e^{-\frac{x}{c_{1}}}}{c_{1}^{b_{1}+1}\Gamma \left(b_{1}+1\right)},$$ (15a)

$$F_{Z}\left(x\right)\approx \frac{\gamma \left(b_{1}+1,c_{1}^{-1}x\right)}{\Gamma \left(b_{1}+1\right)}\approx 1-\frac{\Gamma \left(b_{1}+1,c_{1}^{-1}x\right)}{\Gamma \left(b_{1}+1\right)},$$ (15b)

where $b_{1}=\frac{N{\left[E\left\{Z_{n}\right\}\right]}^{2}}{D\left\{Z_{n}\right\}}\;-\;1$, $c_{1}=\frac{D\left\{Z_{n}\right\}}{E\left\{Z_{n}\right\}}$, γ(.,.) is the lower incomplete Gamma function and Γ(.,.) is the upper incomplete Gamma function.

First, by substituting (15b) and (14b) into (12), $A_{1}$ can be obtained as

$$A_{1}=\left[1-e^{-\frac{\mu_{0}\rho_{Q}}{{\bar{\rho }}_{b}}}\sum_{t=0}^{m_{0}-1}\frac{\mu_{0}^{t}\rho_{Q}^{t}}{t!{\bar{\rho }}_{b}^{t}}\right]\frac{\Gamma \left(b_{1}+1,c_{1}^{-1}\sqrt{\frac{\phi_{\max}d_{1}}{{\bar{\rho }}_{b}}}\right)}{\Gamma \left(b_{1}+1\right)}.$$ (16)

Finally, substituting (15b) and (14b) into (13), $A_{2}$ is written as

$$A_{2}=\frac{\mu_{0}^{m_{0}}}{\Gamma \left(m_{0}\right)\Gamma \left(b_{1}+1\right)}\int_{\frac{\rho_{Q}}{{\bar{\rho }}_{b}}}^{\infty }x^{m_{0}-1}e^{-\mu_{0}x}\Gamma \left(b_{1}+1,\vartheta_{1}\sqrt{x}\right)dx,$$ (17)

where $\vartheta_{1}=c_{1}^{-1}\sqrt{\frac{\phi_{\max}d_{1}}{\rho_{Q}}}$. Based on (17), using $t=\sqrt{x}$, $A_{2}$ can be calculated as

$$A_{2}=\frac{2\mu_{0}^{m_{0}}}{\Gamma \left(m_{0}\right)\Gamma \left(b_{1}+1\right)}\int_{\sqrt{\frac{\rho_{Q}}{{\bar{\rho }}_{b}}}}^{\infty }t^{2m_{0}-1}e^{-\mu_{0}t^{2}}\Gamma \left(b_{1}+1,\vartheta_{1}t\right)dt\;=\frac{2\mu_{0}^{m_{0}}}{\Gamma \left(m_{0}\right)\Gamma \left(b_{1}+1\right)}\int_{0}^{\infty }t^{2m_{0}-1}e^{-\mu_{0}t^{2}}H\left(\sqrt{\frac{{\bar{\rho }}_{b}}{\rho_{Q}}}t-1\right)\;\times \Gamma \left(b_{1}+1,\vartheta_{1}t\right)dt,$$ (18)

where H(.) is heaviside step function. To solve the integral $A_{2}$, we utilize the following transformations involving the Meijer G-function [73, Chpt. 8.4] , [71]

$$e^{-\mu_{0}t^{2}}=G_{0,1}^{1,0}\left(\mu_{0}t^{2}|\begin{array}{c} {}*20c-\\ 0 \end{array}\right),$$ (19a)

$$H\left(\sqrt{\frac{{\bar{\rho }}_{b}}{\rho_{Q}}}t-1\right)=G_{1,1}^{0,1}\left(\sqrt{\frac{{\bar{\rho }}_{b}}{\rho_{Q}}}t|\begin{array}{c} {}*20c1\\ 0 \end{array}\right),$$ (19b)

$$\Gamma \left(b_{1}+1,\vartheta_{1}t\right)=G_{1,2}^{2,0}\left(\vartheta_{1}t|\begin{array}{c} {}*20c1\\ b_{1}+1,0 \end{array}\right),$$ (19c)

Plugging (19c), (19b) and (19a) into (18), $A_{2}$ can be expressed as

$$A_{2}=\frac{2\mu_{0}^{m_{0}}}{\Gamma \left(m_{0}\right)\Gamma \left(b_{1}+1\right)}\int_{0}^{\infty }t^{2m_{0}-1}G_{0,1}^{1,0}\left(\mu_{0}t^{2}|\begin{array}{c} {}*20c-\\ 0 \end{array}\right)\;\times G_{1,1}^{0,1}\left(\sqrt{\frac{{\bar{\rho }}_{b}}{\rho_{Q}}}t|\begin{array}{c} {}*20c1\\ 0 \end{array}\right)G_{1,2}^{2,0}\left(\vartheta_{1}t|\begin{array}{c} {}*20c1\\ b_{1}+1,0 \end{array}\right)dt.$$ (20)

We apply the following (21) to solve integrals (20) in which $H_{p,q:u,v:e,f}^{m,n:s,t:i,j}\left(\cdot \right)$ stands for the extended generalized bivariate Fox H-function (EGBFHF) in [74]. This function can be conveniently evaluated using mathematical software such as Mathematica [75, Table I] and Matlab [76, Appx A].

$$\int_{0}^{\infty }x^{\lambda -1}G_{p,q}^{m,0}\left(\eta x|\begin{array}{c} {𝐚}_{p}\\ {𝐛}_{q} \end{array}\right)G_{p_{2},q_{2}}^{m_{2},n_{2}}\left(\theta x^{h}|\begin{array}{c} {𝐜}_{p_{2}}\\ {𝐝}_{q_{2}} \end{array}\right)G_{p_{3},q_{3}}^{m_{3},n_{3}}\left(\delta x^{k}|\begin{array}{c} {𝐞}_{p_{3}}\\ {𝐟}_{q_{3}} \end{array}\right)dx\;=\eta^{-\lambda }H_{q,p:p_{2},q_{2}:p_{3},q_{3}}^{0,m:m_{2},n_{2}:m_{3},n_{3}}\left(\begin{array}{c} \left(1-{𝐛}_{q}-\lambda ;h,k\right)\\ \left(1-{𝐚}_{p}-\lambda ;h,k\right) \end{array}|\begin{array}{c} \left({𝐜}_{p_{2}},1\right)\\ \left({𝐝}_{q_{2}},1\right) \end{array}|\begin{array}{c} \left({𝐞}_{p_{3}},1\right)\\ \left({𝐟}_{q_{3}},1\right) \end{array}|\frac{\theta }{\eta^{h}},\frac{\delta }{\eta^{k}}\right).\;$$ (21)

Base on (21), $A_{2}$ is written as

$$A_{2}=\frac{2\mu_{0}^{m_{0}}}{\Gamma \left(m_{0}\right)\Gamma \left(b_{1}+1\right)}\int_{0}^{\infty }t^{2m_{0}-1}G_{1,2}^{2,0}\left(\vartheta_{1}t|\begin{array}{c} {}*20c1\\ b_{1}+1,0 \end{array}\right)\;\times G_{0,1}^{1,0}\left(\mu_{0}t^{2}|\begin{array}{c} {}*20c-\\ 0 \end{array}\right)G_{1,1}^{0,1}\left(\sqrt{\frac{{\bar{\rho }}_{b}}{\rho_{Q}}}t|\begin{array}{c} {}*20c1\\ 0 \end{array}\right)dt\;=\frac{2\mu_{0}^{m_{0}}\vartheta_{1}^{-2m_{0}}}{\Gamma \left(m_{0}\right)\Gamma \left(b_{1}+1\right)}\Lambda \left(\frac{\mu_{0}}{\vartheta_{1}^{2}},\frac{1}{\vartheta_{1}}\sqrt{\frac{{\bar{\rho }}_{b}}{\rho_{Q}}}\right).$$ (22)

where Λ(a,b,c) is already given below (9). Substituting (22) and (16) into (11), we can obtain (8).

When ξ=0, the closed-form OP of *U*_1_ for PRIS-NOMA may be stated as

$$\begin{array}{c} P_{U_{1}}^{PRIS}=1-\left[1-e^{-\frac{\mu_{0}\rho_{Q}}{{\bar{\rho }}_{b}}}\sum_{t=0}^{m_{0}-1}\frac{\mu_{0}^{t}\rho_{Q}^{t}}{t!{\bar{\rho }}_{b}^{t}}\right]\frac{\Gamma \left(b_{1}+1,c_{1}^{-1}\sqrt{{\bar{\rho }}_{b}^{-1}{\bar{\phi }}_{\max}}\right)}{\Gamma \left(b_{1}+1\right)}\\ -\frac{2\mu_{0}^{m_{0}}{\bar{\vartheta }}_{1}^{-2m_{0}}}{\Gamma \left(m_{0}\right)\Gamma \left(b_{1}+1\right)}\Lambda \left(b_{1},\frac{\mu_{0}}{{\bar{\vartheta }}_{1}^{2}},\frac{1}{{\bar{\vartheta }}_{1}}\sqrt{\frac{{\bar{\rho }}_{b}}{\rho_{Q}}}\right), \end{array}$$ (23)

where ${\bar{\phi }}_{2}=\frac{\gamma_{th_{2}}}{a_{2}-\gamma_{th_{2}}a_{1}}$, ${\bar{\phi }}_{1}=\frac{\gamma_{th_{1}}}{a_{1}}$, ${\bar{\phi }}_{\max}=\max\left({\bar{\phi }}_{1},{\bar{\phi }}_{2}\right)$ and ${\bar{\vartheta }}_{1}=c_{1}^{-1}\sqrt{\frac{{\bar{\phi }}_{\max}}{\rho_{Q}}}$.

### 3.2 The outage probability of *U*_2_

Concerning *U*_2_ facing deteriorated channel conditions, an interruption is initiated if it cannot recognize or decode its transmitted message *x*_2_. In such circumstances, the OP for *U*_2_ within the context of ARIS-NOMA spectrum-sharing networks is defined by

$$P_{U_{2}}^{ARIS}=\mathrm{Pr}\left(\gamma_{x_{2}}<\gamma_{th_{2}}\right)\;=1-\mathrm{Pr}\left(\gamma_{x_{2}}>\gamma_{th_{2}}\right)\;=1-B_{1}-B_{2},$$ (24)

where $B_{1}$ and $B_{1}$ are already given below

$$B_{1}=\mathrm{Pr}\left(\begin{array}{c} {\left|g_{2}^{H}\Theta g_{0}\right|}^{2}>\frac{\phi_{2}}{{\bar{\rho }}_{b}}\left(\xi \beta N_{tn}{\left|g_{2}^{H}\Theta \right|}^{2}+1\right),\\ {\left|h_{0}\right|}^{2}<\frac{\rho_{Q}}{{\bar{\rho }}_{b}} \end{array}\right),$$ (25a)

$$B_{2}=\mathrm{Pr}\left(\begin{array}{c} {\left|g_{2}^{H}\Theta g_{0}\right|}^{2}>\frac{\phi_{2}{\left|h_{0}\right|}^{2}}{\rho_{Q}}\left(\xi \beta N_{tn}{\left|g_{2}^{H}\Theta \right|}^{2}+1\right),\\ {\left|h_{0}\right|}^{2}>\frac{\rho_{Q}}{{\bar{\rho }}_{b}} \end{array}\right).$$ (25b)

Similarly, by solving $P_{U_{1}}^{ARIS}$ and the closed-form of the OP for *U*_2_ within ARIS-NOMA spectrum-sharing networks may be articulated as

$$\begin{array}{c} P_{U_{2}}^{ARIS}=1-\left[1-e^{-\frac{\mu_{0}\rho_{Q}}{{\bar{\rho }}_{b}}}\sum_{t=0}^{m_{0}-1}\frac{\mu_{0}^{t}\rho_{Q}^{t}}{t!{\bar{\rho }}_{b}^{t}}\right]\frac{\Gamma \left(b_{2}+1,c_{2}^{-1}\sqrt{\frac{\phi_{2}d_{2}}{{\bar{\rho }}_{b}}}\right)}{\Gamma \left(b_{2}+1\right)}\\ -\frac{2\mu_{0}^{m_{0}}\vartheta_{2}^{-2m_{0}}}{\Gamma \left(m_{0}\right)\Gamma \left(b_{2}+1\right)}\Lambda \left(b_{2},\frac{\mu_{0}}{\vartheta_{2}^{2}},\frac{1}{\vartheta_{2}}\sqrt{\frac{{\bar{\rho }}_{b}}{\rho_{Q}}}\right), \end{array}$$ (26)

where $d_{2}=\xi \beta N_{tn}N\lambda_{g_{2}}\;+\;1$, $\vartheta_{2}=c_{2}^{-1}\sqrt{\frac{\phi_{2}d_{2}}{\rho_{Q}}}$, $b_{2}=\frac{N\mu_{2}^{2}}{\Omega_{2}}\;-\;1$, $c_{2}=\frac{\Omega_{2}}{\mu_{2}}$, $\mu_{2}=\frac{\Gamma \left(m_{g_{0}}+0.5\right)\Gamma \left(m_{g_{2}}+0.5\right)\sqrt{\lambda_{g_{0}}\lambda_{g_{2}}}}{\Gamma \left(m_{g_{0}}\right)\Gamma \left(m_{g_{2}}\right)\sqrt{m_{g_{0}}m_{g_{2}}}}$, $\Omega_{2}=\lambda_{g_{0}}\lambda_{g_{2}}\left\langle 1-\frac{1}{m_{g_{0}}m_{g_{2}}}{\left[\frac{\Gamma \left(m_{g_{0}}+0.5\right)\Gamma \left(m_{g_{2}}+0.5\right)}{\Gamma \left(m_{g_{0}}\right)\Gamma \left(m_{g_{2}}\right)}\right]}^{2}\right\rangle$.

When parameter ξ is set to zero, the closed-form OP of *U*_2_ for PRIS-NOMA may be stated as

$$\begin{array}{c} P_{U_{2}}^{PRIS}=1-\left[1-e^{-\frac{\mu_{0}\rho_{Q}}{{\bar{\rho }}_{b}}}\sum_{t=0}^{m_{0}-1}\frac{\mu_{0}^{t}\rho_{Q}^{t}}{t!{\bar{\rho }}_{b}^{t}}\right]\frac{\Gamma \left(b_{2}+1,c_{2}^{-1}\sqrt{{\bar{\rho }}_{b}^{-1}{\bar{\phi }}_{2}}\right)}{\Gamma \left(b_{2}+1\right)}\\ -\frac{2\mu_{0}^{m_{0}}{\bar{\vartheta }}_{2}^{-2m_{0}}}{\Gamma \left(m_{0}\right)\Gamma \left(b_{2}+1\right)}\Lambda \left(b_{2},\frac{\mu_{0}}{{\bar{\vartheta }}_{2}^{2}},\frac{1}{{\bar{\vartheta }}_{2}}\sqrt{\frac{{\bar{\rho }}_{b}}{\rho_{Q}}}\right), \end{array}$$ (27)

where ${\bar{\vartheta }}_{2}=c_{2}^{-1}\sqrt{\frac{{\bar{\phi }}_{2}}{\rho_{Q}}}$.

### 3.3 The outage probability of user *O*

In a manner similar to the aforementioned analytical advancements, the probability of outage for orthogonal user *O* within ARIS-OMA spectrum-sharing networks is articulated as by

$$P_{O}^{ARIS}=1-\mathrm{Pr}\left(\gamma_{O}>\gamma_{th_{O}}\right).$$ (28)

where $\gamma_{th_{O}}$ denotes the target SNRs of user *O*.

Subject to the constraints of cascade Nakagami-*m* fading channels, the OP associated with user *O* within ARIS-OMA spectrum-sharing networks may be articulated as

$$\begin{array}{c} P_{O}^{ARIS}=1-\left[1-e^{-\frac{\mu_{0}\rho_{Q}}{{\bar{\rho }}_{b}}}\sum_{t=0}^{m_{0}-1}\frac{\mu_{0}^{t}\rho_{Q}^{t}}{t!{\bar{\rho }}_{b}^{t}}\right]\frac{\Gamma \left(b_{O}+1,c_{O}^{-1}\sqrt{\frac{\phi_{O}d_{O}}{{\bar{\rho }}_{b}}}\right)}{\Gamma \left(b_{O}+1\right)}\\ -\frac{2\mu_{0}^{m_{0}}\vartheta_{O}^{-2m_{0}}}{\Gamma \left(m_{0}\right)\Gamma \left(b_{O}+1\right)}\Lambda \left(b_{O},\frac{\mu_{0}}{\vartheta_{O}^{2}},\frac{1}{\vartheta_{O}}\sqrt{\frac{{\bar{\rho }}_{b}}{\rho_{Q}}}\right), \end{array}$$ (29)

where $\phi_{O}=\frac{\gamma_{th_{O}}}{\beta }$, $d_{O}=\xi \beta N_{tn}\lambda_{O}+1$ and $\vartheta_{O}=c_{O}^{-1}\sqrt{\frac{\phi_{O}d_{O}}{\rho_{Q}}}$.

When the parameter ξ is set to be zero, the closed-form OP of user *O* for PRIS-OMA can be expressed as

$$\begin{array}{c} P_{O}^{PRIS}=1-\left[1-e^{-\frac{\mu_{0}\rho_{Q}}{{\bar{\rho }}_{b}}}\sum_{t=0}^{m_{0}-1}\frac{\mu_{0}^{t}\rho_{Q}^{t}}{t!{\bar{\rho }}_{b}^{t}}\right]\frac{\Gamma \left(b_{O}+1,c_{O}^{-1}\sqrt{\frac{\gamma_{th_{O}}}{{\bar{\rho }}_{b}}}\right)}{\Gamma \left(b_{O}+1\right)}\\ -\frac{2\mu_{0}^{m_{0}}{\bar{\vartheta }}_{O}^{-2m_{0}}}{\Gamma \left(m_{0}\right)\Gamma \left(b_{O}+1\right)}\Lambda \left(b_{O},\frac{\mu_{0}}{{\bar{\vartheta }}_{O}^{2}},\frac{1}{{\bar{\vartheta }}_{O}}\sqrt{\frac{{\bar{\rho }}_{b}}{\rho_{Q}}}\right), \end{array}$$ (30)

where ${\bar{\vartheta }}_{O}=c_{O}^{-1}\sqrt{\frac{\gamma_{th_{O}}}{\rho_{Q}}}$.

### 3.4 Diversity analysis

In this segment, we select the diversity order to scrutinize the outage characteristics of ARIS-NOMA spectrum-sharing networks. In other terms, the diversity order serves to characterize the rate at which the likelihood of outage diminishes as the SNR increases [77]. As the diversity order is enhanced, the probability of outage declines more rapidly. To elaborate further, the diversity order is articulated as

$$D=-\underset{{\bar{\rho }}_{b}\to \infty }{\lim}\frac{\log\left(P^{\infty }\left({\bar{\rho }}_{b}\right)\right)}{\log\left({\bar{\rho }}_{b}\right)}.$$ (31)

where $P^{\infty }\left({\bar{\rho }}_{b}\right)$ represents the asymptotic OP in case of high SNRs.

When ${\bar{\rho }}_{b}$ goes to infinity then we have $A_{1}\approx 0$ and $\frac{\rho_{Q}}{{\bar{\rho }}_{b}}\approx 0$, the asymptotic expression for $P_{U_{1}}^{ARIS,\infty }$ is calculated as:

$$P_{U_{1}}^{ARIS,\infty }=\mathrm{Pr}\left(\left|\sum_{n=1}^{N}g_{0}^{n}g_{1}^{n}\right|>\sqrt{\frac{\phi_{\max}d_{1}{\left|h_{0}\right|}^{2}}{\rho_{Q}}},{\left|h_{0}\right|}^{2}>0\right)\;=1-\frac{\mu_{0}^{m_{0}}}{\Gamma \left(m_{0}\right)\Gamma \left(b_{1}+1\right)}\int_{0}^{\infty }x^{m_{0}-1}e^{-\mu_{0}x}\Gamma \left(b_{1}+1,\vartheta_{1}\sqrt{x}\right)dx.$$ (32)

Let $t=\sqrt{x}$ and rewrite the exponential term with the G-function, (32) is rewritten as follows

$$P_{U_{1}}^{ARIS,\infty }=1-\frac{2\mu_{0}^{m_{0}}}{\Gamma \left(m_{0}\right)\Gamma \left(b_{1}+1\right)}\int_{0}^{\infty }t^{2m_{0}-1}e^{-\mu_{0}t^{2}}\Gamma \left(b_{1}+1,\vartheta_{1}t\right)dt\;=1-\frac{2\mu_{0}^{m_{0}}}{\Gamma \left(m_{0}\right)\Gamma \left(b_{1}+1\right)}\int_{0}^{\infty }t^{2m_{0}-1}\;\times G_{1,2}^{2,0}\left(\vartheta_{1}t|\begin{array}{c} {}*20c1\\ b_{1}+1,0 \end{array}\right)G_{0,1}^{1,0}\left(\mu_{0}t^{2}|\begin{array}{c} {}*20c-\\ 0 \end{array}\right)dt.$$ (33)

Using property [78, Eq. 2.24], the asymptotic OP of *U*_1_ for ARIS-NOMA may be calculated as follows:

$$P_{U_{1}}^{ARIS,\infty }=1-\frac{2^{b_{1}+0.5+2m_{0}}\mu_{0}^{m_{0}}\vartheta_{1}^{-2m_{0}}}{\sqrt{2\pi }\Gamma \left(m_{0}\right)\Gamma \left(b_{1}+1\right)}\;\times G_{4,3}^{1,4}\left(\frac{4\mu_{0}}{\vartheta_{1}^{2}}|\begin{array}{c} {}*20c\frac{-b_{1}-2m_{0}}{2},\frac{1-b_{1}-2m_{0}}{2},\frac{1-2m_{0}}{2},1-m_{0}\\ 0,-m_{0},\frac{1-2m_{0}}{2} \end{array}\right).$$ (34)

When ξ equals zero, the asymptotic OP of *U*_1_ for PRIS-NOMA may be calculated as:

$$P_{U_{1}}^{PRIS,\infty }=1-\frac{2^{b_{1}+0.5+2m_{0}}\mu_{0}^{m_{0}}{\bar{\vartheta }}_{1}^{-2m_{0}}}{\sqrt{2\pi }\Gamma \left(m_{0}\right)\Gamma \left(b_{1}+1\right)}\;\times G_{4,3}^{1,4}\left(\frac{4\mu_{0}}{{\bar{\vartheta }}_{1}^{2}}|\begin{array}{c} {}*20c\frac{-b_{1}-2m_{0}}{2},\frac{1-b_{1}-2m_{0}}{2},\frac{1-2m_{0}}{2},1-m_{0}\\ 0,-m_{0},\frac{1-2m_{0}}{2} \end{array}\right).$$ (35)

As ${\bar{\rho }}_{b}$ approaches infinity, the asymptotic OP of *U*_2_ for ARIS/PRIS-NOMA may be calculated by

$$P_{U_{2}}^{ARIS,\infty }=1-\frac{2^{b_{1}+0.5+2m_{0}}\mu_{0}^{m_{0}}\vartheta_{2}^{-2m_{0}}}{\sqrt{2\pi }\Gamma \left(m_{0}\right)\Gamma \left(b_{1}+1\right)}\;\times G_{4,3}^{1,4}\left(\frac{4\mu_{0}}{\vartheta_{2}^{2}}|\begin{array}{c} {}*20c\frac{-b_{1}-2m_{0}}{2},\frac{1-b_{1}-2m_{0}}{2},\frac{1-2m_{0}}{2},1-m_{0}\\ 0,-m_{0},\frac{1-2m_{0}}{2} \end{array}\right),$$ (36)

and

$$P_{U_{2}}^{PRIS,\infty }=1-\frac{2^{b_{1}+0.5+2m_{0}}\mu_{0}^{m_{0}}{\bar{\vartheta }}_{2}^{-2m_{0}}}{\sqrt{2\pi }\Gamma \left(m_{0}\right)\Gamma \left(b_{1}+1\right)}\;\times G_{4,3}^{1,4}\left(\frac{4\mu_{0}}{{\bar{\vartheta }}_{2}^{2}}|\begin{array}{c} {}*20c\frac{-b_{1}-2m_{0}}{2},\frac{1-b_{1}-2m_{0}}{2},\frac{1-2m_{0}}{2},1-m_{0}\\ 0,-m_{0},\frac{1-2m_{0}}{2} \end{array}\right).$$ (37)

Similarly, by solving (36) and (37), the asymptotic OP of user *O* for ARIS-OMA and PRIS-OMA can be separately expressed as

$$P_{O}^{ARIS,\infty }=1-\frac{2^{b_{1}+0.5+2m_{0}}\mu_{0}^{m_{0}}\vartheta_{O}^{-2m_{0}}}{\sqrt{2\pi }\Gamma \left(m_{0}\right)\Gamma \left(b_{O}+1\right)}\;\times G_{4,3}^{1,4}\left(\frac{4\mu_{0}}{\vartheta_{O}^{2}}|\begin{array}{c} {}*20c\frac{-b_{O}-2m_{0}}{2},\frac{1-b_{O}-2m_{0}}{2},\frac{1-2m_{0}}{2},1-m_{0}\\ 0,-m_{0},\frac{1-2m_{0}}{2} \end{array}\right),$$ (38)

and

$$P_{O}^{PRIS,\infty }=1-\frac{2^{b_{1}+0.5+2m_{0}}\mu_{0}^{m_{0}}{\bar{\vartheta }}_{O}^{-2m_{0}}}{\sqrt{2\pi }\Gamma \left(m_{0}\right)\Gamma \left(b_{O}+1\right)}\;\times G_{4,3}^{1,4}\left(\frac{4\mu_{0}}{{\bar{\vartheta }}_{O}^{2}}|\begin{array}{c} {}*20c\frac{-b_{O}-2m_{0}}{2},\frac{1-b_{O}-2m_{0}}{2},\frac{1-2m_{0}}{2},1-m_{0}\\ 0,-m_{0},\frac{1-2m_{0}}{2} \end{array}\right).$$ (39)

Upon substituting equations (39), (38), (37), (36), (35), and (34) into equation (31), it is observed that the diversity order of user *Z*, $Z\in \left\{U_{1},U_{2},O\right\}$ is equivalent to zero. This phenomenon arises from the residual interference induced by the temperature constraint at the PU. We see that diversity order is the power exponent, which defines the rate of convergence of PU to the error floor. Furthermore, it can be deduced that the diversity order is linked to the number of reflected elements and the order of the channels, and it is noteworthy that the introduction of thermal noise from the ARIS does not influence the diversity order.

### 3.5 Delay-limited transmission

In delay-limited systems, the BS transmits data at a consistent rate, which is affected by the stochastic variations of the wireless channel. The resultant system throughput can be expressed as

$$\tau_{dlt}^{⋆}=\left(1-P_{U_{1}}^{⋆}\right)R_{1}+\left(1-P_{U_{2}}^{⋆}\right)R_{2},⋆\in \left\{ARIS,PRIS\right\}$$ (40)

## 4 Ergodic data rate

In this segment, the performance of the ergodic data rate (EDR) for each device can be assessed in both ARIS and PRIS scenarios. Unlike [11], our objective is to derive an approximate expression for EDR. Both the OP and EDR can be validated using well-known software tools like Mathematica or Matlab. Essentially, EDR is defined as the long-term average data rate that can be achieved without factoring in any delay constraints. The EDR constitutes a significant metric for assessing the efficacy of a communication system. When the conditions of the channel dictate the user’s desired rate, the ergodic data rate may be articulated as the optimal rate at which the system is capable of transmitting accurately across cascade Nakagami-*m* fading channels, and it can be delineated as

$$\begin{array}{cc} {}*20cC=𝔼\left[\log_{2}\left(1+\gamma_{\epsilon }\right)\right] & ,\epsilon \in \left\{x_{1},x_{2},O\right\} \end{array}$$ (41)

### 4.1 The ergodic data rate of ARIS networks

The average user *g*, $\left(g\in \left\{U_{1},U_{2},O\right\}\right)$ attainable rate can be extracted from (41), characterized as $C_{g}^{ARIS}=𝔼\left[\log_{2}\left(1+\gamma_{\epsilon }\right)\right]$. In this scenario, the formulation of user *g* for ARIS-NOMA networks can be allocated in the subsequent proposition.

Based on cascade Nakagami-*m* fading channels, the EDR of *U*_1_ for ARIS-NOMA networks may be estimated as (42), in which *K* is a complexity-accuracy tradeoff parameter, $\nu =\left[1-e^{-\frac{\mu_{0}\rho_{Q}}{{\bar{\rho }}_{b}}}\sum_{t=0}^{m_{0}-1}{\left(t!\right)}^{-1}{\left(\frac{\mu_{0}\rho_{Q}}{{\bar{\rho }}_{b}}\right)}^{t}\right]$, $G\left(x\right)=\tan\left(\frac{\pi \left(x+1\right)}{4}\right)$, ${℘}_{1}\left(x\right)=c_{1}^{-1}\sqrt{\frac{d_{1}G\left(x\right)}{\beta a_{1}\rho_{Q}}}$, $\sec^{2}\left(x\right)=1/\cos^{2}\left(x\right)$ and $\Phi_{k}=\cos\left(\frac{2k-1}{2K}\pi \right)$.

$$C_{U_{1}}^{ARIS}\approx \frac{\pi^{2}}{4K\Gamma \left(b_{1}+1\right)\ln2}\sum_{k=1}^{K}\sqrt{1-\Phi_{k}^{2}}\sec^{2}\left(\frac{\pi \left(\Phi_{k}+1\right)}{4}\right)\frac{1}{1+G\left(\Phi_{k}\right)}\;\times [\nu \Gamma \left(b_{1}+1,c_{1}^{-1}\sqrt{\frac{d_{1}G\left(\Phi_{k}\right)}{{\bar{\rho }}_{b}\beta a_{1}}}\right)+\frac{2\mu_{0}^{m_{0}}{\left[{℘}_{1}\left(\Phi_{k}\right)\right]}^{-2m_{0}}}{\Gamma \left(m_{0}\right)}\;\times \Lambda \left(b_{1},\frac{\mu_{0}}{{\left[{℘}_{1}\left(\Phi_{k}\right)\right]}^{2}},\frac{1}{{℘}_{1}\left(\Phi_{k}\right)}\sqrt{\frac{{\bar{\rho }}_{b}}{\rho_{Q}}}\right)],\;$$ (42)

Using the coherent phase shifting technique and changing (4) into (41) further, the EDR for *U*_1_ of ARIS-NOMA is computed by

$$C_{U_{1}}^{ARIS}=𝔼\left[\log_{2}\left(1+\gamma_{x_{1}}\right)\right]\;=𝔼\left[\log_{2}\left(1+\underset{X}{\underbrace{\frac{\beta \rho_{b}a_{1}{\left|g_{1}^{H}\Theta g_{0}\right|}^{2}}{\xi \beta N_{tn}{\left|g_{1}^{H}\Theta \right|}^{2}+1}}}\right)\right]\;=\frac{1}{\ln2}\int_{0}^{\infty }\frac{1-F_{X}\left(x\right)}{1+x}dx.$$ (43)

Hence, $F_{X}\left(x\right)$ is calculated by

$$F_{X}\left(x\right)=1-\left[V_{1}+V_{2}\right],$$ (44)

where $V_{1}=\mathrm{Pr}\left({\left|\sum_{n=1}^{N}g_{0}^{n}g_{1}^{n}\right|}^{2}>\frac{d_{1}y}{{\bar{\rho }}_{b}\beta a_{1}},{\left|h_{0}\right|}^{2}<\frac{\rho_{Q}}{{\bar{\rho }}_{b}}\right)$ and $V_{2}=\mathrm{Pr}\left({\left|\sum_{n=1}^{N}g_{0}^{n}g_{1}^{n}\right|}^{2}>\frac{d_{1}{\left|h_{0}\right|}^{2}y}{\rho_{Q}\beta a_{1}},{\left|h_{0}\right|}^{2}>\frac{\rho_{Q}}{{\bar{\rho }}_{b}}\right)$.

The equation is presented in (44) can be readily derived as obtained in (11). Hence, we can rewrite $F_{X}\left(x\right)$ as

$$F_{X}\left(x\right)=1-\frac{\nu }{\Gamma \left(b_{1}+1\right)}\Gamma \left(b_{1}+1,c_{1}^{-1}\sqrt{\frac{d_{1}x}{{\bar{\rho }}_{b}\beta a_{1}}}\right)\;-\frac{2\mu_{0}^{m_{0}}{\left[{\hat{\vartheta }}_{1}\left(x\right)\right]}^{-2m_{0}}}{\Gamma \left(m_{0}\right)\Gamma \left(b_{1}+1\right)}\Lambda \left(b_{1},\frac{\mu_{0}}{{\left[{\hat{\vartheta }}_{1}\left(x\right)\right]}^{2}},\frac{1}{{\hat{\vartheta }}_{1}\left(x\right)}\sqrt{\frac{{\bar{\rho }}_{b}}{\rho_{Q}}}\right),$$ (45)

where $\nu =\left[1-e^{-\frac{\mu_{0}\rho_{Q}}{{\bar{\rho }}_{b}}}\sum_{t=0}^{m_{0}-1}{\left(t!\right)}^{-1}{\left(\frac{\mu_{0}\rho_{Q}}{{\bar{\rho }}_{b}}\right)}^{t}\right]$ and ${\hat{\vartheta }}_{1}\left(x\right)=c_{1}^{-1}\sqrt{\frac{d_{1}x}{\beta a_{1}\rho_{Q}}}$.

Substituting (45) into (43), we have $C_{U_{1}}^{ARIS}$ is given by

$$C_{U_{1}}^{ARIS}=\frac{1}{\Gamma \left(b_{1}+1\right)\ln2}\int_{0}^{\infty }\frac{1}{1+x}[\nu \Gamma \left(b_{1}+1,c_{1}^{-1}\sqrt{\frac{d_{1}x}{{\bar{\rho }}_{b}\beta a_{1}}}\right)\;+\frac{2\mu_{0}^{m_{0}}{\left[{\hat{\vartheta }}_{1}\left(x\right)\right]}^{-2m_{0}}}{\Gamma \left(m_{0}\right)}\Lambda \left(b_{1},\frac{\mu_{0}}{{\left[{\hat{\vartheta }}_{1}\left(x\right)\right]}^{2}},\frac{1}{{\hat{\vartheta }}_{1}\left(x\right)}\sqrt{\frac{{\bar{\rho }}_{b}}{\rho_{Q}}}\right)]dx.$$ (46)

Exchanging the variable $t=\frac{4}{\pi }\arctan\left(x\right)-1\Rightarrow \tan\left(\frac{\pi \left(t+1\right)}{4}\right)=x\Rightarrow \frac{\pi }{4}\sec^{2}\left(\frac{\pi }{4}\left(t+1\right)\right)dt=dx$, we have $C_{U_{1}}^{ARIS}$ can be calculated as (47),

$$C_{U_{1}}^{ARIS}=\frac{\pi }{4\Gamma \left(b_{1}+1\right)\ln2}\int_{-1}^{1}\frac{\sec^{2}\left(\frac{\pi }{4}\left(t+1\right)\right)}{1+G\left(t\right)}\left[\nu \Gamma \left(b_{1}+1,c_{1}^{-1}\sqrt{\frac{d_{1}G\left(t\right)}{{\bar{\rho }}_{b}\beta a_{1}}}\right)\;+\frac{2\mu_{0}^{m_{0}}{\left[{℘}_{1}\left(t\right)\right]}^{-2m_{0}}}{\Gamma \left(m_{0}\right)}\;\times \Lambda \left(b_{1},\frac{\mu_{0}}{{\left[{℘}_{1}\left(t\right)\right]}^{2}},\frac{1}{{℘}_{1}\left(t\right)}\sqrt{\frac{{\bar{\rho }}_{b}}{\rho_{Q}}}\right)\;\right]dt,$$ (47)

where $G\left(t\right)=\tan\left(\frac{\pi \left(t+1\right)}{4}\right)$, ${℘}_{1}\left(t\right)=c_{1}^{-1}\sqrt{\frac{d_{1}G\left(t\right)}{\beta a_{1}\rho_{Q}}}$ and $\sec^{2}\left(x\right)=1/\cos^{2}\left(x\right)$.

Unfortunately, finding a closed-form expression for (47) is a tough task, but an accurate approximation can be obtained for it. By using Gaussian-Chebyshev quadrature [79, Eq. 25.4.38], it can be achieved in (42), in which $\Phi_{k}=\cos\left(\frac{2k-1}{2K}\pi \right)$.

Given cascade Nakagami-*m* fading channels, the ergodic data rate of *U*_2_ in ARIS-NOMA networks is approximated as (48) in which $\Xi \left(t\right)=\frac{t}{\beta \rho_{b}\left(a_{2}-a_{1}t\right)}$, $\Psi \left(t\right)=\frac{a_{2}\left(t+1\right)}{2a_{1}}$, ${℘}_{2}\left(t\right)=c_{2}^{-1}\sqrt{\frac{d_{2}\Xi \left(\Psi \left(t\right)\right)}{\rho_{Q}}}$ and $\Phi_{k}=\cos\left(\frac{2k-1}{2K}\pi \right)$.

$$C_{U_{2}}^{ARIS}\approx \frac{\pi a_{2}}{2Ka_{1}\ln2}\sum_{k=1}^{K}\frac{\sqrt{1-\Phi_{k}^{2}}}{1+\Psi \left(\Phi_{k}\right)}\;\times \left[\frac{\nu }{\Gamma \left(b_{2}+1\right)}\Gamma \left(b_{2}+1,c_{2}^{-1}\sqrt{\frac{d_{2}\Xi \left(\Psi \left(\Phi_{k}\right)\right)}{{\bar{\rho }}_{b}}}\right)\;-\frac{2\mu_{0}^{m_{0}}{\left[{℘}_{2}\left(\Phi_{k}\right)\right]}^{-2m_{0}}}{\Gamma \left(m_{0}\right)\Gamma \left(b_{2}+1\right)}\Lambda \left(b_{2},\frac{\mu_{0}}{{\left[{℘}_{2}\left(\Phi_{k}\right)\right]}^{2}},\frac{1}{{℘}_{2}\left(\Phi_{k}\right)}\sqrt{\frac{{\bar{\rho }}_{b}}{\rho_{Q}}}\right)\;\right],$$ (48)

By plugging (5) into (41), the EDR for *U*_2_ of ARIS-NOMA networks can be calculated by

$$C_{U_{2}}^{ARIS}=𝔼\left[\log_{2}\left(1+\gamma_{x_{2}}\right)\right]\;=\frac{1}{\ln2}\int_{0}^{a_{2}/a_{1}}\frac{1}{1+x}{\bar{F}}_{Y}\left(\frac{x}{\beta \rho_{b}\left(a_{2}-a_{1}x\right)}\right)dx,$$ (49)

where ${\bar{F}}_{Y}\left(x\right)$ denotes the complementary CDF, ${\bar{F}}_{Y}\left(x\right)=1-F_{Y}\left(x\right)$ and

$$Y=\frac{\beta \rho_{b}a_{2}{\left|\sum_{n=1}^{N}g_{0}^{n}g_{2}^{n}\right|}^{2}}{\beta \rho_{b}a_{1}{\left|\sum_{n=1}^{N}g_{0}^{n}g_{2}^{n}\right|}^{2}+d_{2}}.$$ (50)

We have $F_{Y}\left(x\right)$ may be easily deduced as gained in (26). So $F_{Y}\left(x\right)$ may be written as

$$F_{Y}\left(x\right)=1-\frac{\nu }{\Gamma \left(b_{2}+1\right)}\Gamma \left(b_{2}+1,c_{2}^{-1}\sqrt{\frac{d_{2}x}{{\bar{\rho }}_{b}}}\right)\;-\frac{2\mu_{0}^{m_{0}}{\left[{\hat{\vartheta }}_{2}\left(x\right)\right]}^{-2m_{0}}}{\Gamma \left(m_{0}\right)\Gamma \left(b_{2}+1\right)}\Lambda \left(b_{2},\frac{\mu_{0}}{{\left[{\hat{\vartheta }}_{2}\left(x\right)\right]}^{2}},\frac{1}{{\hat{\vartheta }}_{2}\left(x\right)}\sqrt{\frac{{\bar{\rho }}_{b}}{\rho_{Q}}}\right),$$ (51)

where ${\hat{\vartheta }}_{2}\left(x\right)=c_{2}^{-1}\sqrt{\frac{d_{2}x}{\rho_{Q}}}$.

Substituting the result of (51) into (49), the EDR of *U*_2_ is given by (52)

$$C_{U_{2}}^{ARIS}\overset{\left(a\right)}{=}\frac{a_{2}}{2a_{1}\ln2}\int_{-1}^{1}\frac{1}{1+\Psi \left(t\right)}\left[\frac{\nu }{\Gamma \left(b_{2}+1\right)}\Gamma \left(b_{2}+1,c_{2}^{-1}\sqrt{\frac{d_{2}\Xi \left(\Psi \left(t\right)\right)}{{\bar{\rho }}_{b}}}\right)\;-\frac{2\mu_{0}^{m_{0}}{\left[{℘}_{2}\left(t\right)\right]}^{-2m_{0}}}{\Gamma \left(m_{0}\right)\Gamma \left(b_{2}+1\right)}\Lambda \left(b_{2},\frac{\mu_{0}}{{\left[{℘}_{2}\left(t\right)\right]}^{2}},\frac{1}{{℘}_{2}\left(t\right)}\sqrt{\frac{{\bar{\rho }}_{b}}{\rho_{Q}}}\right)\;\right],\;C_{U_{2}}^{ARIS}\overset{\left(b\right)}{\approx }\frac{\pi a_{2}}{2Ka_{1}\ln2}\sum_{k=1}^{K}\frac{\sqrt{1-\Phi_{k}^{2}}}{1+\Psi \left(\Phi_{k}\right)}\frac{\nu }{\Gamma \left(b_{2}+1\right)}\Gamma \left(b_{2}+1,c_{2}^{-1}\sqrt{\frac{d_{2}\Xi \left(\Psi \left(\Phi_{k}\right)\right)}{{\bar{\rho }}_{b}}}\right)\;-\frac{2\mu_{0}^{m_{0}}{\left[{℘}_{2}\left(\Phi_{k}\right)\right]}^{-2m_{0}}}{\Gamma \left(m_{0}\right)\Gamma \left(b_{2}+1\right)}\Lambda \left(b_{2},\frac{\mu_{0}}{{\left[{℘}_{2}\left(\Phi_{k}\right)\right]}^{2}},\frac{1}{{℘}_{2}\left(\Phi_{k}\right)}\sqrt{\frac{{\bar{\rho }}_{b}}{\rho_{Q}}}\right),\;$$ (52)

Then, (52) can be re-written, where step (a) follows by letting $t=\frac{2a_{1}x}{a_{2}}\;-\;1$; step (b) follows by using Gaussian-Chebyshev quadrature approximation; *K* is a parameter which determines the trade-off between complexity and accuracy; $\Xi \left(t\right)=\frac{t}{\beta \rho_{b}\left(a_{2}-a_{1}t\right)}$, $\Psi \left(t\right)=\frac{a_{2}\left(t+1\right)}{2a_{1}}$, ${℘}_{2}\left(t\right)=c_{2}^{-1}\sqrt{\frac{d_{2}\Xi \left(\Psi \left(t\right)\right)}{\rho_{Q}}}$ and $\Phi_{k}=\cos\left(\frac{2k-1}{2K}\pi \right)$.

The EDR of the orthogonal user *O* in ARIS-OMA networks may be estimated as (53), in which ${℘}_{O}\left(x\right)=c_{O}^{-1}\sqrt{\frac{d_{O}G\left(x\right)}{\rho_{Q}\beta }}$.

$$C_{O}^{ARIS}\approx \frac{\pi^{2}}{4K\Gamma \left(b_{O}+1\right)\ln2}\sum_{k=1}^{K}\sqrt{1-\Phi_{k}^{2}}\sec^{2}\left(\frac{\pi \left(\Phi_{k}+1\right)}{4}\right)\frac{1}{1+G\left(\Phi_{k}\right)}\;\times [\nu \Gamma \left(b_{O}+1,c_{O}^{-1}\sqrt{\frac{d_{O}G\left(\Phi_{k}\right)}{{\bar{\rho }}_{b}\beta }}\right)\;+\frac{2\mu_{0}^{m_{0}}{\left[{℘}_{O}\left(\Phi_{k}\right)\right]}^{-2m_{0}}}{\Gamma \left(m_{0}\right)}\Lambda \left(b_{O},\frac{\mu_{0}}{{\left[{℘}_{O}\left(\Phi_{k}\right)\right]}^{2}},\frac{1}{{℘}_{O}\left(\Phi_{k}\right)}\sqrt{\frac{{\bar{\rho }}_{b}}{\rho_{Q}}}\right)],$$ (53)

### 4.2 The ergodic data rate of PRIS networks

The EDR of PRIS-NOMA networks may be calculated for the exceptional case (ξ=0), as the reflecting element for the PRIS does not contain amplification.

For ξ=0, the EDR of *U*_1_ for PRIS-NOMA across cascade Nakagami-*m* fading channels may be calculated as (54), in which ${\bar{℘}}_{1}\left(t\right)=c_{1}^{-1}\sqrt{\frac{G\left(t\right)}{a_{1}\rho_{Q}}}$.

$$C_{U_{1}}^{PRIS}\approx \frac{\pi^{2}}{4K\Gamma \left(b_{1}+1\right)\ln2}\sum_{k=1}^{K}\sqrt{1-\Phi_{k}^{2}}\sec^{2}\left(\frac{\pi \left(\Phi_{k}+1\right)}{4}\right)\frac{1}{1+G\left(\Phi_{k}\right)}\;\times \left[\nu \Gamma \left(b_{1}+1,c_{1}^{-1}\sqrt{\frac{G\left(\Phi_{k}\right)}{{\bar{\rho }}_{b}a_{1}}}\right)+\frac{2\mu_{0}^{m_{0}}{\left[{\bar{℘}}_{1}\left(\Phi_{k}\right)\right]}^{-2m_{0}}}{\Gamma \left(m_{0}\right)}\Lambda \left(b_{1},\frac{\mu_{0}}{{\left[{\bar{℘}}_{1}\left(\Phi_{k}\right)\right]}^{2}},\frac{1}{{\bar{℘}}_{1}\left(\Phi_{k}\right)}\sqrt{\frac{{\bar{\rho }}_{b}}{\rho_{Q}}}\right)\right],\;$$ (54)

When ξ=0, the EDR of *U*_2_ for PRIS-NOMA-HIS across cascade Nakagami-m fading channels is approximated as (55) in which $\bar{\Xi }\left(x\right)=\frac{x}{\rho_{b}\left(a_{2}-a_{1}x\right)}$ and ${\bar{℘}}_{2}\left(t\right)=c_{2}^{-1}\sqrt{\frac{d_{2}\bar{\Xi }\left(\Psi \left(t\right)\right)}{\rho_{Q}}}$.

$$C_{U_{2}}^{PRIS}\approx \frac{\pi a_{2}}{2Ka_{1}\ln2}\sum_{k=1}^{K}\frac{\sqrt{1-\Phi_{k}^{2}}}{1+\Psi \left(\Phi_{k}\right)}\;\times \left[\frac{\nu }{\Gamma \left(b_{2}+1\right)}\Gamma \left(b_{2}+1,c_{2}^{-1}\sqrt{\frac{d_{2}\bar{\Xi }\left(\Psi \left(\Phi_{k}\right)\right)}{{\bar{\rho }}_{b}}}\right)\;-\frac{2\mu_{0}^{m_{0}}{\left[{\bar{℘}}_{2}\left(\Phi_{k}\right)\right]}^{-2m_{0}}}{\Gamma \left(m_{0}\right)\Gamma \left(b_{2}+1\right)}\Lambda \left(b_{2},\frac{\mu_{0}}{{\left[{\bar{℘}}_{2}\left(\Phi_{k}\right)\right]}^{2}},\frac{1}{{\bar{℘}}_{2}\left(\Phi_{k}\right)}\sqrt{\frac{{\bar{\rho }}_{b}}{\rho_{Q}}}\right)\;\right],$$ (55)

The EDR of the orthogonal user *O* in PRIS-OMA networks is given as (56) in which ${\bar{℘}}_{O}\left(x\right)=c_{O}^{-1}\sqrt{\frac{d_{O}G\left(x\right)}{\rho_{Q}}}$.

$$C_{O}^{PRIS}\approx \frac{\pi^{2}}{4K\Gamma \left(b_{O}+1\right)\ln2}\sum_{k=1}^{K}\sqrt{1-\Phi_{k}^{2}}\sec^{2}\left(\frac{\pi \left(\Phi_{k}+1\right)}{4}\right)\frac{1}{1+G\left(\Phi_{k}\right)}\;\times [\nu \Gamma \left(b_{O}+1,c_{O}^{-1}\sqrt{\frac{d_{O}G\left(\Phi_{k}\right)}{{\bar{\rho }}_{b}}}\right)+\frac{2\mu_{0}^{m_{0}}{\left[{\bar{℘}}_{O}\left(\Phi_{k}\right)\right]}^{-2m_{0}}}{\Gamma \left(m_{0}\right)}\;\times \Lambda \left(b_{O},\frac{\mu_{0}}{{\left[{\bar{℘}}_{O}\left(\Phi_{k}\right)\right]}^{2}},\frac{1}{{\bar{℘}}_{O}\left(\Phi_{k}\right)}\sqrt{\frac{{\bar{\rho }}_{b}}{\rho_{Q}}}\right)],\;$$ (56)

### 4.3 Delay-tolerate transmission

In delay-tolerant frameworks, the BS disseminates data at a consistent rate determined by the constraints of the user’s channel condition, utilizing the EDR as a maximum threshold. Consequently, the system throughput for ARIS/PRIS-NOMA networks may be articulated as

$$\tau_{dtt}^{⋆}=C_{U_{1}}^{⋆}+C_{U_{2}}^{⋆},⋆\in \left\{ARIS,PRIS\right\}.$$ (57)

## 5 Energy efficiency

In ARIS-NOMA networks, the overall power usage of the system mainly includes the power transmitted from the BS, the power used by the BS itself, the power utilized by ARIS, the output power from ARIS, and the power consumption of the hardware at the user devices. This can be expressed precisely by [59,64]

$${\bar{P}}_{total}=\ell^{-1}{\bar{P}}_{b}+{\bar{P}}_{BS}+N{\bar{P}}_{RE}+{\bar{P}}_{out}+\sum_{i=1}^{2}{\bar{P}}_{U_{i}},$$ (58)

where ℓ represents the efficiency of the transmitter power amplifier, ${\bar{P}}_{b}$ signifies the power emitted at the BS. ${\bar{P}}_{BS}$ refers to the overall static power loss at the BS, while $N{\bar{P}}_{RE}$ denotes the cumulative hardware static power loss from *N* reflector elements at the ARIS. ${\bar{P}}_{out}$ indicates the output power from the ARIS, and ${\bar{P}}_{U_{i}}$ represents the hardware power consumed by the *i*th user. It can be concluded that the energy efficiency of the system can be expressed as the proportion of the sum rate to the overall power usage of the system, which is denoted as

$$\eta_{EE,\Delta }^{⋆}=\frac{\tau_{\Delta }^{⋆}}{{\bar{P}}_{total}},$$ (59)

where ⋆∈{ARIS,PRIS} and Δ∈{dlt,dtt}. $\tau_{dlt}^{⋆}$ and $\tau_{dtt}^{⋆}$ can be derived from (40) and (57), respectively.

## 6 Optimizing power allocation in the ARIS-assisted NOMA spectrum-sharing systems

In this segment, we examine the optimization of power distribution to enhance the efficacy of the proposed ARIS-assisted NOMA spectrum-sharing framework. Initially, we ascertain the optimal power allocation coefficients at the BS (referred to as $a_{1}^{*}$) with the objective of individually minimizing the OP within the ARIS-NOMA network. This endeavor concentrates on determining the power allocation coefficients of $a_{1}^{*}$ at the BS that yield the minimal OP. To accomplish this, we establish the ensuing minimization problems:

$$\underset{a_{1}}{\min}P_{U_{i}}^{⋆}\left(a_{1}\right)\;s.t.\{\begin{array}{c} a_{1}+a_{2}=1\\ 0<a_{1}<1 \end{array}$$ (60)

Here, ⋆∈{ARIS,PRIS} and i∈{1,2}.

The objective function delineated in (60) exhibits a joint concavity in *a*_1_, as illustrated in Fig 6. In light of the derived OP expressions, it proves challenging to ascertain closed-form expressions for the optimal values of the power allocation factor *a*_1_. Fortunately, one may employ low-complexity algorithms founded on the golden section search method to address this issue. For instance, in Algorithm 1, we delineate the procedures to ascertain the precise value of $a_{1}^{*}$ that minimizes the OP for the second user. The accuracy of Algorithm 1 is predominantly contingent upon the specified step search interval *Δ*.

**Fig 2:**
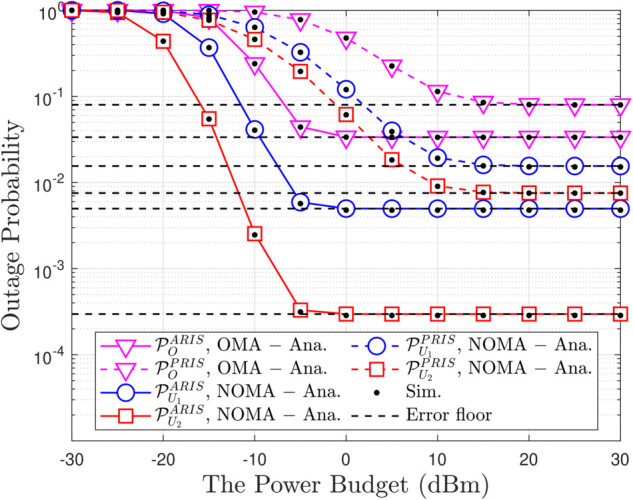
Outage probability versus the transmit power budget, with N = 5, β = 5, m = 1, $\rho_{Q}$ = 5 [dBm] and $R_{1}$ = $R_{2}$ = 1.5 [BPCU].

**Fig 3:**
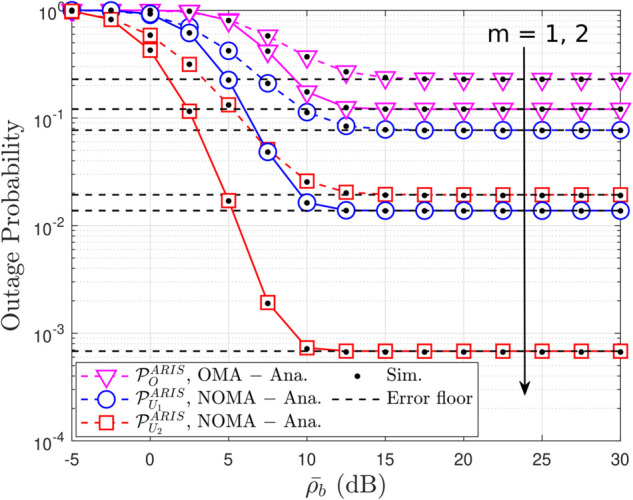
Outage probability versus the transmit power ${\bar{\rho }}_{b}$, with N = 4 and $\rho_{Q}$ = 5 [dB].

**Fig 4:**
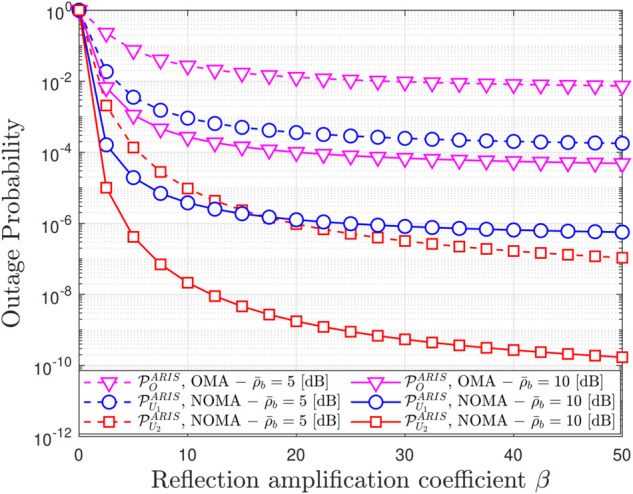
Outage probability versus the reflection amplitude factors β, with N = 8, m = 1, $\rho_{Q}$ = 10 [dB] and $R_{1}$ = $R_{2}$ = 1.5 [BPCU].

**Fig 5:**
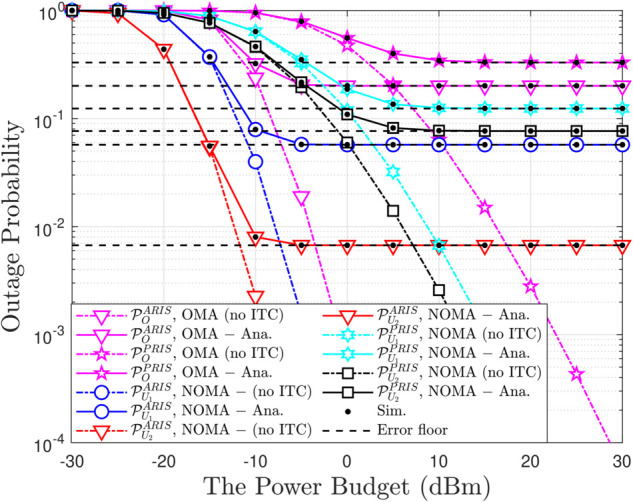
Outage probability versus the transmit power budget, with N = 5, m = 1 and $\rho_{Q}$ = 5 [dBm].

**Algorithm 1. Optimization Algorithm to find $a_{1}^{*}$ based on Golden section search** [71].



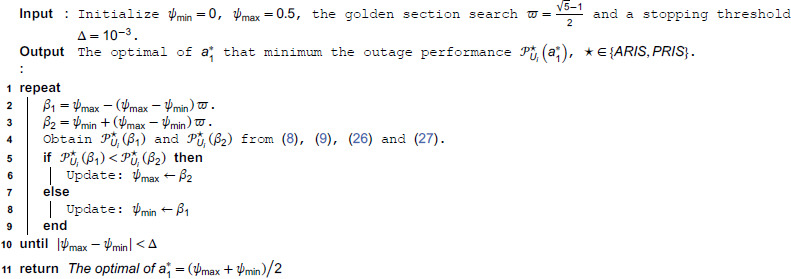



## 7 Numerical results

In this section, we conduct a numerical assessment of our theoretical findings regarding the performance of the OP. We establish the fading parameters as $m=m_{g_{0}}=m_{g_{1}}=m_{g_{2}}=m_{h_{0}}$. The results obtained from Monte Carlo simulations are averaged over 10^6^ independent trials. The target rate is measured in bits per channel user and is succinctly referred to as BPCU. In the subsequent figures, we abbreviate “Ana." and “Sim." to represent analytical computation and Monte Carlo simulation, respectively. The other principal parameters are encapsulated in Table 2.

**Table 2:** Main parameters for our simulations [59].

Parameters	Notation	Values
Monte Carlo simulations	–	10^6^ iterations
Power splitting factors	$\left\{a_{1},a_{2}\right\}$	{0.2,0.8}
Target rates SINR to decode *x*_1_ and *x*_2_	$\left\{R_{1},R_{2}\right\}$	{1.5,1} BPCU
The fading parameter	*m*	1
The interference constraint at PU	$\rho_{Q}$	5 dB
The reflection coefficient of ARIS	*β*	5
The thermal noise power at ARIS	*N*_*tn*_	–20 dB
The hardware power consumption	$\left\{{\bar{P}}_{SW},{\bar{P}}_{DC}\right\}$	{−5,−10} dBm
Distance from BS to PU	*d*_*PU*_	6 m
Distance from BS to ARIS	*d*_*ARIS*_	5 m
Distance from ARIS to *U*_1_	$d_{U_{1}}$	5 m
Distance from ARIS to *U*_2_	$d_{U_{2}}$	10 m
The pathloss exponent	ς	2
The power distribution factor	*ρ*	0.5

It is important to clarify that in order to validate the accuracy of the outcomes, we have ensured that the total power used by PRIS-NOMA and ARIS-NOMA remains constant. To elaborate, we set the total power consumption for two active and passive systems as ${\bar{P}}_{b,total}^{ARIS}={\bar{P}}_{BS}^{ARIS}+{\bar{P}}_{RIS}^{ARIS}+N\left({\bar{P}}_{SW}+{\bar{P}}_{DC}\right)$, ${\bar{P}}_{b,total}^{PRIS}={\bar{P}}_{BS}^{PRIS}+N{\bar{P}}_{SW}$, and ${\bar{P}}_{b}={\bar{P}}_{b,total}^{ARIS}={\bar{P}}_{b,total}^{PRIS}$, where ${\bar{P}}_{BS}^{ARIS}=\varrho ({\bar{P}}_{b,total}^{ARIS}-N\left({\bar{P}}_{SW}+{\bar{P}}_{DC}\right))$ and ${\bar{P}}_{BS}^{PRIS}=(1-\varrho )({\bar{P}}_{b,total}^{ARIS}-N\left({\bar{P}}_{SW}+{\bar{P}}_{DC}\right))$ represent the transmission power of the base station in ARIS and PRIS networks, respectively. Meanwhile, ϱ∈[0,1] denotes the power distribution factor, ${\bar{P}}_{SW}$ denotes the power consumed by the control circuit and phase shift switches for each RIS element, and ${\bar{P}}_{DC}$ refers to the direct current biasing power [61,80].

Fig 2 provides a comprehensive overview of the relationship between OP, a key measure of connection reliability, and the average transmit power (${\bar{\rho }}_{b}$). As expected, the OP curve sharply decreases as ${\bar{\rho }}_{b}$ increases, reaffirming a fundamental principle in wireless communication: a stronger signal is more resilient to noise, thus reducing the likelihood of disconnection. However, the key takeaway here is not just this general trend but the stark contrast in performance between ARIS-NOMA and PRIS-NOMA at the same transmit power level ARIS-NOMA consistently achieves a lower OP than PRIS-NOMA, particularly in the low transmit power regime.This highlights the superior efficiency of ARIS, with its active signal amplification capabilities, in leveraging the available transmit power to bolster connection reliability. In essence, ARIS-NOMA acts as a skillful “resource allocator," effectively utilizing every “unit" of energy to fortify the stability of the connection.

Fig 3 illustrates the OP performance of the ARIS-assisted NOMA and OMA systems under varying channel conditions and transmit power levels. It is evident from the graph that the ARIS-NOMA system consistently outperforms the ARIS-OMA system across all scenarios. This observation underscores the superior performance of NOMA in mitigating interference and improving spectral efficiency compared to OMA, particularly in challenging channel conditions. And the increasing ${\bar{\rho }}_{b}$ also improved OP, as in a similar discussion in Fig 2. Moreover, the OP is plotted against the Nakagami-*m* fading parameter, which characterizes the severity of fading in the wireless channel. The graph also reveals that the OP is significantly affected by the severity of fading. A higher value of m indicates a less severe fading environment. For both NOMA and OMA, the OP is notably higher when m=1 (Rayleigh fading) compared to when m=2, indicating that fading has a detrimental impact on system performance. This observation highlights the importance of employing techniques to combat fading, such as ARIS, to maintain reliable communication links.

Fig 4 illustrates the impact of the reflection amplification coefficient (*β*) on OP for both ARIS-OMA and ARIS-NOMA. It is observed that OP decreases as *β* increases, but only up to a certain point. After that, OP reaches a steady state, and further increasing *β* does not provide significant improvement. Specifically, with *β* increasing from 0 to 10, OP is significantly reduced. However, when *β* increases from 10 to 50, OP decreases only slightly and remains almost constant. This observation indicates the existence of an optimal *β* value where OP reaches its minimum. Increasing *β* beyond this optimal value does not yield significant performance benefits and may lead to unnecessary energy consumption. Therefore, selecting the optimal *β* is crucial to balancing OP reduction and energy saving.

A thorough examination of OP for both OMA and NOMA schemes under different ITC at the PU is shown in Fig 5. One important finding is that, with or without ITC, NOMA consistently outperforms OMA, demonstrating its higher spectrum efficiency and user fairness as a result of its capacity to support numerous users within the same time-frequency resources. The impact of ITC on OP is also obviously shown in the image. Both OMA and NOMA experience a decrease in OP in the absence of ITC because a stricter interference limitation reduces secondary users’ transmit power and raises their OP. ${\bar{\rho }}_{b}$ from 15 dB to 25 dB lowers the OP more than the other plan, particularly in the scheme without ITC, indicating the important influence of ITC. The analytical expression obtained in equation (1) of the study, which clearly depicts the connection between ${\bar{\rho }}_{b}$ and ITC value, supports these facts. In conclusion, Fig 5 provides a comprehensive overview of the outage performance of ARIS-assisted OMA and NOMA schemes under ITC scenarios, highlighting the influence of ITC on system performance and the superiority of NOMA.

**Fig 6:**
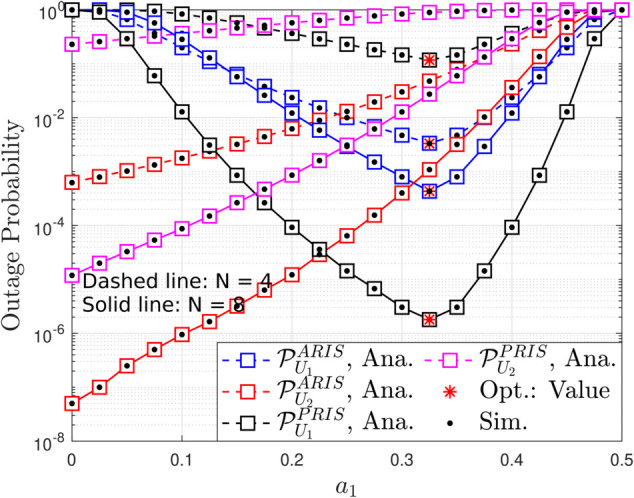
Outage probability versus power allocation factors, with m = 2, $R_{1}$ = $R_{2}$ = 1 [BPCU] and $\rho_{Q}$ = 5 [dB].

Fig 6 provides a detailed analysis of the OP as a function of the power allocation factor (*a*_1_) in both ARIS-NOMA and PRIS-NOMA systems. The analysis considers two scenarios with different numbers of reflecting elements (*N* = 4 and *N* = 8) for both ARIS and PRIS. The figure clearly demonstrates the impact of power allocation on the outage performance of both the primary user (*U*_1_) and the secondary user (*U*_2_) in both ARIS and PRIS systems. A striking observation is the existence of an optimal *a*_1_ (*a*_1_ = 0.325) value that minimizes the OP for both users. This optimal point signifies the ideal balance in power distribution between the users, minimizing their outage probabilities while adhering to the interference constraints. This close alignment of optimal *a*_1_ values for both users highlights the effectiveness of NOMA in achieving a fair and efficient resource allocation. Comparing ARIS and PRIS, we observe that ARIS consistently outperforms PRIS in terms of OP for both users across all values of *a*_1_. This advantage stems from the active signal amplification capability of ARIS, which effectively boosts the received signal strength and reduces the OP. Furthermore, increasing the number of reflecting elements from *N* = 4 to *N* = 8 leads to a noticeable improvement in OP for both users in both ARIS and PRIS. This improvement is attributed to the enhanced spatial diversity offered by a larger number of reflecting elements, which helps to mitigate the detrimental effects of fading. The results emphasize the importance of optimizing power allocation to minimize OP and demonstrate the performance gains achieved by ARIS and a larger number of reflecting elements.

**Fig 7:**
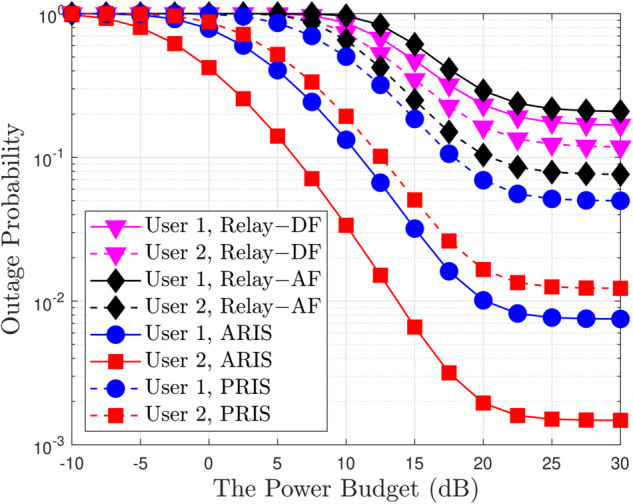
Outage probability versus the transmit power budget in Relay link and ARIS/PRIS link, with N = 2, m = 1, $R_{1}$ = $R_{2}$ = 1 [BPCU] and $\rho_{Q}$ = 10 [dB].

**Fig 8:**
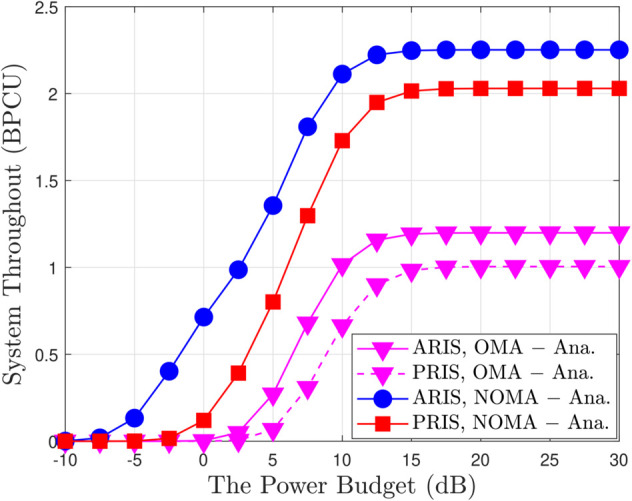
System throughput versus the transmit power budget, with m = 1, $a_{1}$ = 0.1, $a_{2}$ = 0.9 and $\rho_{Q}$ = 5 [dB].

**Fig 9:**
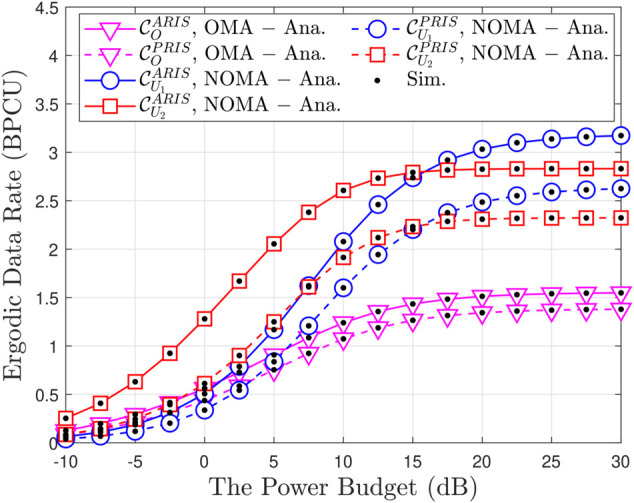
Ergodic data rate versus the transmit power ${\bar{\rho }}_{b}$, with N = 5, m = 1, $a_{1}$ = 0.1, $a_{2}$ = 0.9 and $\rho_{Q}$ = 5 [dB].

Fig 7 plots the outage comparison between ARIS/PRIS and the relaying scheme, including the DF and AF cases. We can observe that the performance of ARIS is better than the other schemes. Since the RIS can enhance the channel quality and improve the received SINR of users by increasing the number of reflecting elements. Fig 8 provides a comprehensive analysis of system throughput (ST) as it relates to transmit power (${\bar{\rho }}_{b}$) for various multiple access schemes, including both OMA and NOMA, implemented with both ARIS and PRIS. A key observation is the consistent superiority of NOMA over OMA in terms of ST across the entire range of transmit power for both ARIS and PRIS, highlighting NOMA’s enhanced spectral efficiency due to its ability to accommodate multiple users within the same time-frequency resource block. Furthermore, ARIS consistently outperforms PRIS for both OMA and NOMA due to its active signal amplification capabilities, which boost received signal strength and enhance overall throughput. As transmit power increases, ST initially increases for all schemes but eventually saturates due to the interference constraint at the primary user and inherent channel capacity limits. For example, at a transmit power of 10 dB, ARIS-NOMA achieves an ST of approximately 2.8 BPCU, while PRIS-NOMA achieves around 2.5 BPCU, demonstrating the throughput gain provided by ARIS. The analytical results, represented by solid and dashed lines, closely match the simulation results, validating the accuracy of the derived expressions for system throughput, such as equation (33) in the paper, which explicitly captures the relationship between ST, transmit power, power allocation factors, and the type of RIS. Fig 9 provides a detailed illustration of how the EDR changes with varying transmit power (${\bar{\rho }}_{b}$) in a wireless communication system employing both OMA and NOMA schemes; the analysis considers both ARIS and PRIS configurations. A prominent trend observed in the figure is the positive correlation between transmit power and EDR. As ${\bar{\rho }}_{b}$ increases, the EDR also increases for all considered schemes (ARIS-OMA, PRIS-OMA, ARIS-NOMA, and PRIS-NOMA). This behavior is intuitive, as higher transmit power translates to a stronger signal, leading to a higher achievable data rate. However, the rate of EDR growth varies across different schemes and exhibits a saturation effect at higher transmit power levels. Specifically, NOMA schemes (both ARIS and PRIS) demonstrate a steeper initial rise in EDR compared to their OMA counterparts, highlighting NOMA’s superior spectral efficiency in accommodating multiple users within the same time-frequency resources. Furthermore, ARIS consistently outperforms PRIS for both OMA and NOMA, showcasing the benefits of active signal amplification in enhancing data rates. This advantage is particularly noticeable in the lower transmit power regime, where ARIS exhibits a more pronounced improvement in EDR compared to PRIS. As the transmit power increases beyond a certain threshold, the EDR growth gradually slows down and eventually saturates for all schemes. In summary, the results highlight the positive correlation between transmit power and EDR, the superior performance of NOMA over OMA, the benefits of ARIS over PRIS, and the saturation effect observed at high transmit power levels due to interference constraints and channel capacity limitations.

**Fig 10:**
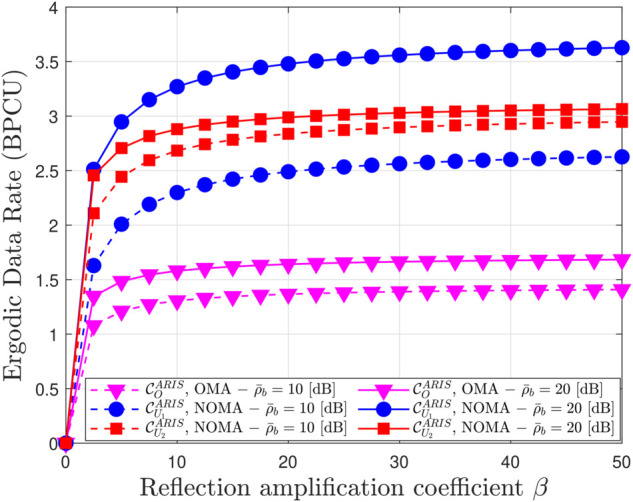
Ergodic data rate versus the reflection amplitude factors β, with N = 4, m = 1 and $\rho_{Q}$ = 5 [dB].

**Fig 11:**
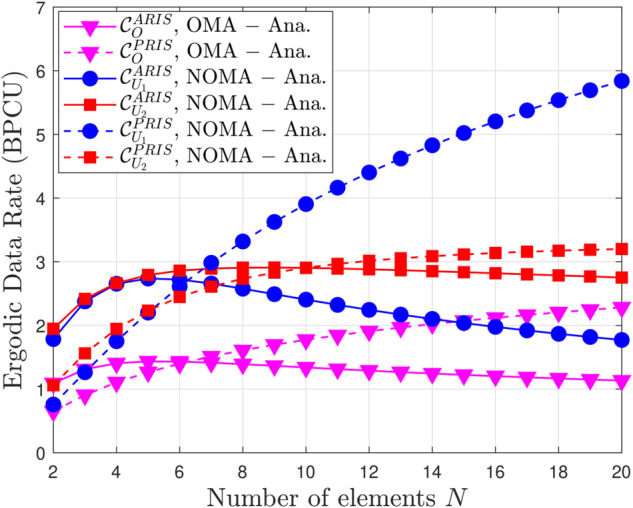
Ergodic data rate versus the number of elements N, with β = 5, m = 1 and power budget = 15 [dB].

Figs 10 and 11 provide a comprehensive analysis of the factors influencing the EDR in ARIS and PRIS-assisted OMA and NOMA systems. Fig 10 illustrates the impact of the reflection amplification coefficient (*β*) on EDR, demonstrating that EDR increases with *β* but eventually saturates, highlighting the importance of *β* optimization. ARIS consistently achieves higher EDR than PRIS due to active signal amplification, while NOMA outperforms OMA due to superior spectral efficiency. Higher transmit power also leads to higher EDR, as shown by the different curves for ${\bar{\rho }}_{b}=10$ dB and ${\bar{\rho }}_{b}=20$ dB. For example, with ARIS-NOMA at ${\bar{\rho }}_{b}=10$ dB, increasing *β* from 0 to 10 significantly boosts EDR, but further increasing *β* to 50 yields only marginal gains, demonstrating the saturation effect. Fig 10 further explores the impact of the number of reflecting elements (*N*) on EDR. Similar to the trend observed with *β*, EDR increases with *N* but eventually saturates, indicating that there is an optimal number of elements for maximizing EEDR. Again, ARIS consistently outperforms PRIS, and NOMA outperforms OMA, across all values of N. These observations are supported by analytical expressions in the paper, which capture the relationship between EDR, *β*, transmit power, the number of reflecting elements, and the type of RRIS.In conclusion, Figs 10 and 11 offer valuable insights into the factors influencing EDR in ARIS and PRIS-assisted communication systems, emphasizing the importance of optimizing system parameters such as *β* and N, and highlighting the advantages of ARIS over PRIS and NOMA over OMA in achieving higher data rates.

**Fig 12:**
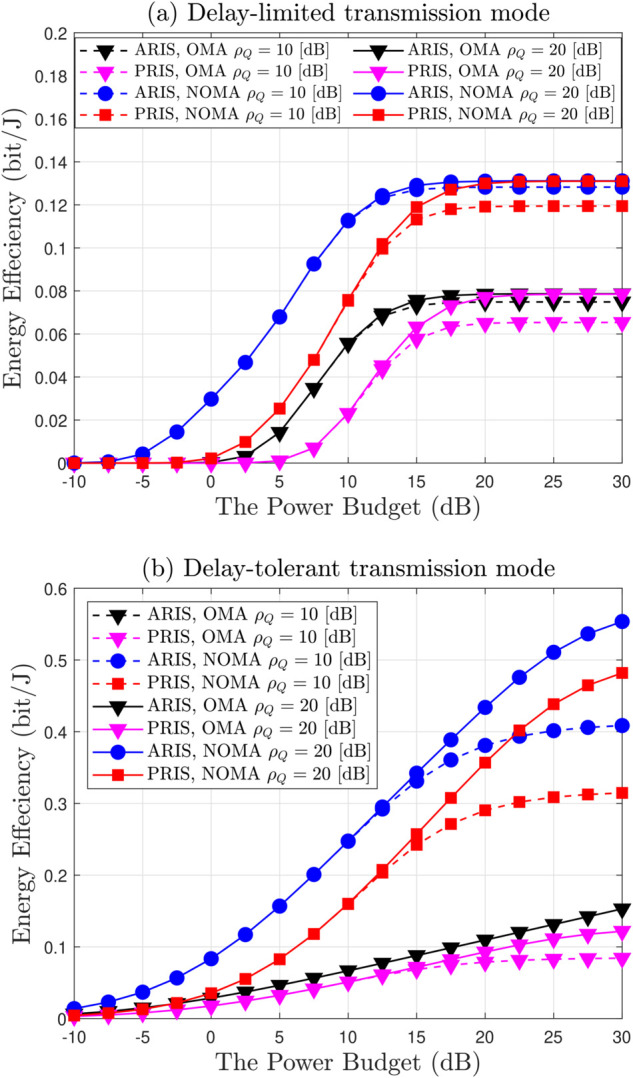
Energy efficiency in delay-limited and delay-tolerant transmission mode, with *N* = 4, *m* = 1, ℓ = 2/3, ${\bar{P}}_{b}$ = 10 dBw, ${\bar{P}}_{BS}$ = 4 dBw, ${\bar{P}}_{RE}$ = 10 dBm, ${\bar{P}}_{out}$ = 10 dBm and ${\bar{P}}_{U_{i}}$ = 10 dBm.

Fig 12 illustrates the energy efficiency graphs in both delay-limited and delay-tolerant transmission modes where *N* = 4, *m* = 0.7, $R_{1}=R_{2}=2$ BPCU, ℓ=2/3, ${\bar{P}}_{b}=10$ dBw, ${\bar{P}}_{BS}=4$ dBw, ${\bar{P}}_{RE}=10$ dBm, ${\bar{P}}_{out}=10$ dBm and ${\bar{P}}_{U_{i}}=10$ dBm [64]. Fig 12(a) depicts the delay-limited transmission mode, where energy efficiency is computed from achievable throughput under the outage constraints metric within the context of ARIS/PRIS-NOMA networks. The influence of interference, quantified by $\rho_{Q}$, is scrutinized for the values of 10 dB and 20 dB. The findings indicate that NOMA consistently surpasses OMA in terms of energy efficiency, whilst ARIS demonstrates superior performance when compared to PRIS. An increase in interference $\rho_{Q}$ enhances efficiency across all configurations, and energy efficiency exhibits a consistent rise with respect to ${\bar{\rho }}_{b}$, indicative of improved signal quality. Fig 12(b) corresponds to the delay-tolerant transmission mode, where energy efficiency is computed from the EDR. It presents a comparison of energy efficiency (bit/J) concerning the transmit power (${\bar{P}}_{b}$) for ARIS and PRIS under the OMA and NOMA schemes, with $\rho_{Q}$ established at 10 dB and 20 dB. The findings suggest that energy efficiency is enhanced as ${\bar{P}}_{b}$ is elevated. NOMA consistently surpasses OMA, thereby illustrating its superior efficiency, while ARIS demonstrates a greater energy efficiency in comparison to PRIS across all evaluated conditions. An increase in $\rho_{Q}$ contributes positively to energy efficiency across all configurations. It is noteworthy that the ARIS-NOMA configuration at $\rho_{Q}=20$ dB attains the optimal performance, highlighting the significant potential inherent in the integration of active intelligent surfaces with advanced communication methodologies for the development of energy-efficient systems. In summary, the ARIS-NOMA arrangement at elevated values of ${\bar{P}}_{b}$ and $\rho_{Q}$ yields the most optimal energy efficiency, underscoring the advantages of advanced technologies and effective interference management.

## 8 Conclusion

This paper investigated the performance of an ARIS-assisted NOMA network in a cognitive spectrum-sharing scenario. By exploiting the ARIS’s ability to intelligently adjust phase shifts and amplify reflected signals, the proposed system mitigates inter-user interference, overcomes multiplicative fading, and enhances signal strength compared with conventional PRIS-assisted NOMA and OMA. We derived exact closed-form expressions for the OP, throughput, and EE, as well as an approximate expression for the EDR, all validated through Monte Carlo simulations. Asymptotic analysis was conducted to reveal the achievable diversity order. Furthermore, we formulated and solved an optimization problem for the NOMA power allocation coefficient to minimize OP, demonstrating additional performance gains. Numerical results showed that the ARIS-assisted NOMA system consistently outperforms both PRIS and OMA counterparts in terms of OP, throughput, EDR, and EE. These findings highlight the ARIS’s potential to significantly enhance spectral efficiency, reliability, and coverage in next-generation spectrum-sharing NOMA networks. Future research could explore the effects of imperfect CSI, advanced resource allocation strategies, and extensions to multi-cell and multi-antenna scenarios.

## Supporting information

S1 File MATLAB_code. (ZIP)
